# A novel pro-oxidant combination of resveratrol and copper reduces transplant related toxicities in patients receiving high dose melphalan for multiple myeloma (RESCU 001)

**DOI:** 10.1371/journal.pone.0262212

**Published:** 2022-02-04

**Authors:** Anshul Agarwal, Aakanksha Khandelwal, Kavita Pal, Naveen Kumar Khare, Vishal Jadhav, Murarilal Gurjar, Sachin Punatar, Anant Gokarn, Avinash Bonda, Lingaraj Nayak, Sadhana Kannan, Vikram Gota, Navin Khattry, Indraneel Mittra

**Affiliations:** 1 Bone Marrow Transplant Unit, Department of Medical Oncology, Tata Memorial Centre, Advance Centre for Treatment, Research and Education in Cancer, Navi Mumbai, Maharashtra, India; 2 Homi Bhabha National Institute, Mumbai, Maharashtra, India; 3 Translational Research Laboratory, Tata Memorial Centre, Advance Centre for Treatment, Research and Education in Cancer, Navi Mumbai, Maharashtra, India; 4 Clinical Pharmacology Laboratory, Tata Memorial Centre, Advance Centre for Treatment, Research and Education in Cancer, Navi Mumbai, Maharashtra, India; 5 Department of Biostatistics, Tata Memorial Centre, Advance Centre for Treatment, Research and Education in Cancer, Navi Mumbai, Maharashtra, India; National Cancer Institute, UNITED STATES

## Abstract

**Background:**

Transplant related toxicity is a major therapeutic challenge. We have previously reported that the toxicity of chemotherapy is largely not directly because of the drugs themselves; rather it is mainly due to DNA damage, apoptosis and hyper-inflammation triggered by cell-free chromatin particles that are released because of drug-induced host cell death. Cell-free chromatin particles can be inactivated by free-radicals which are generated when the nutraceuticals resveratrol and copper are administered orally. We investigated if a combination of resveratrol and copper would reduce transplant related toxicities in an exploratory, prospective dose-escalation study.

**Patients and methods:**

Twenty-five patients with multiple myeloma were enrolled between March 2017 to August 2019. Patients were divided into 3 groups: control (Group 1, N = 5) received vehicle alone; group 2 (N = 15) received resveratrol-copper at dose level I (resveratrol = 5.6 mg and copper = 560 ng); group 3 (N = 5) received resveratrol-copper at dose level II (resveratrol = 50 mg and copper = 5 μg). The dose was given twice daily with the first dose administered 48 hours before administering melphalan and continued until day +21 post-transplant. Common Terminology Criteria for Adverse Events version 4.02 was used to assess toxicities which included oral mucositis, nausea, vomiting and diarrhea. Measurement of inflammatory cytokines was done by ELISA.

**Results:**

All patients (100%) in the control group developed grade 3/4 oral mucositis compared to 8/20 (40%) in both resveratrol-copper group 2 plus group 3 combined (P = 0.039). Reduction in inflammatory cytokines: salivary TNF - α (p = 0.012) and IL—1β (p = 0.009) in dose level I but not in dose level II was observed.

**Conclusions:**

A combination of resveratrol-copper reduced transplant related toxicities in patients with multiple myeloma receiving high dose melphalan. We conclude that relatively inexpensive nutraceuticals may be useful as adjuncts to chemotherapy to reduce its toxicity.

**Registration:**

The trial was registered under Clinical Trial Registry of India (no.CTRI/2018/02/011905).

## Introduction

Mucosal barrier injury is a frequent complication of intensive conditioning regimen used for hematopoietic stem cell transplantation [1]. The incidence of severe mucositis (WHO grade III/IV) following intensive chemo conditioning range from 10–78% [2–4]. Other common side effects as a result of mucosal barrier injury include nausea, vomiting and diarrhea.

It has been reported that cell-free chromatin (cfCh) particles that are released from the billions of cells that die in the body everyday can integrate into genomes of healthy cells to trigger dsDNA breaks, apoptosis and activation of inflammatory cytokines [5, 6]. We have hypothesized that repeated genomic integration of cfCh particles occurring throughout life may be the underlying cause of ageing and degenerative disorders [7, 8]. We have previously reported that toxicity of chemotherapy is largely not due to the direct effects of the drugs themselves; rather it is mainly due to DNA damage, apoptosis and hyper-inflammation triggered by cell-free chromatin particles that are released because of drug induced host cell death [9]. Cell-free chromatin particles released from initial round of drug-induced cell death triggers a cascading effect of more cell death leading to a vicious cycle of further rounds of DNA damage, apoptosis and hyper-inflammation which perpetuate and amplify the toxic effects of chemotherapy [9]. Cell-free chromatin particles can be inactivated/ degraded by free-radicals that are generated when the nutraceuticals resveratrol and copper are administered orally.

Resveratrol is a nutraceutical plant polyphenol with anti-oxidant properties and has been extensively investigated for its health benefits. Diaz-Gerevini et al. [10] Fukuhara and Miyata [11] were the first to report that resveratrol can act as a pro-oxidant in presence of copper, which is also a nutraceutical and is extensively used as a health supplement [12]. Resveratrol has the property to reduce copper (II) to copper (I) resulting in the generation of highly unstable free-radicals [11] which can degrade / inactivate cell-free chromatin and thereby can prevent chemotherapy related toxicity in mice [9]. The free-radical generating property of resveratrol-copper is retained even when the molar concentration of copper is reduced 10,000 fold [9, 13–15]. This dose of resveratrol -copper (1: 10^−4^) is effective in degrading cell-free chromatin when administered to mice orally twice daily [9, 13, 14]. Therefore, concentrations of resveratrol and copper used in our current study were nearly 50 times and 4000 times less, respectively, than those generally recommended as health supplements {Biotivia [16] Carlson lab [17]}.

Based on the above pre-clinical observations relating to cell-free chromatin induced chemotherapy toxicity, we investigated if a combination of resveratrol and copper would reduce transplant related toxicities in patients receiving high dose melphalan for multiple myeloma in an exploratory, prospective dose-escalation study.

## Methods

This prospective, single centre, dose escalation pilot study was carried out at a large tertiary care cancer centre in India. The study was approved by the Institutional Ethics Committee of Tata Memorial Centre, Mumbai, India, and written informed consent was obtained from all patients. The trial was registered under Clinical Trial Registry of India (no.CTRI/2018/02/011905). Twenty-five patients with multiple myeloma receiving hematopoietic stem cell transplant with high dose melphalan in the bone marrow transplant unit of the institution were recruited in this study. Patients were ≥ 18 years with ECOG performance status ≤ 2 and creatinine clearance of ≥ 50mL/min (by Cockcroft Gault formula). The 25 patients were initially planned for enrollment sequentially in 5 dosing groups of five patients each. Resveratrol and copper were administered twice daily; resveratrol in powder form was administered mixed in honey followed by copper in water solution. The control group (Group 1) received honey alone, 15 mL twice daily; group 2 received resveratrol-copper at dose level I (DL-I; resveratrol = 5.6 mg and copper = 560 ng); groups 3 and 4 were planned to receive resveratrol-copper in multiples of 10, i.e. group 3 (DL-II; resveratrol = 50 mg and copper = 5 μg), and group 4 (DL-III; resveratrol = 500 mg and copper = 0.5 mg). The fifth group (DL-IV) was planned to receive doses of resveratrol -copper that are recommended as health supplement (resveratrol = 500 mg,b.d.and copper = 5 mg, b.d.). The lowest dose, i.e. DL-I, was arrived at by direct conversion of doses employed in our pre-clinical studies using a conventional formula [18].

No improvement was observed with respect to toxicity status between DL-I and DL-II after completion of resveratrol -copper treatment in first 10 patients. Therefore, further dose escalation was not undertaken; instead after obtaining appropriate IRB approval, 10 additional patients were recruited in DL-I. Thus, the study finally comprised 3 groups: control (N = 5); group 2 (DL-I; N = 15) and group3 (DL-II; N = 5).

First doses of honey (in the control group or group-1) and resveratrol and copper (in groups 2 and 3) were administered 48 hours prior to melphalan conditioning and continued thereafter until day +21 post-transplant (counting day of stem cell infusion as day 0). Melphalan at a dose of 200 mg/m^2^ was administered one day prior to stem cell infusion (day-1). Blood (6 mL) and saliva (~10 mL) were collected immediately prior to chemotherapy (day 0) and on days 3, 6, 9, 12, 15, 18 and 21. Inflammatory cytokines IL-6, TNF- α, IL-1β, IL-10, IFN- γ were estimated in blood and saliva by ELISA only in group 1 (control) and first five patients of DL-I (group 2). Adverse events (mucositis, nausea, vomiting and diarrhea) were documented and graded using the Common Terminology Criteria for Adverse Events (CTCAE), version 4.02. Additionally, daily pain score (using visual pain scale), requirement of total parenteral nutrition and requirement of opioids was also recorded. Procurement sources of resveratrol and copper and of ELISA kits are given in the S1 Table.

### Statistical analysis

Statistical comparison among groups for the primary endpoint of incidence of immediate transplant-related toxicities was performed using SPSS statistical software for windows version 20.0 (Armonk, New York, IBM Corp.). Fisher’s exact test was used to calculate significance levels for categorical data. The Area under the Concentration-time Curve (AUC) for serum and salivary cytokines were calculated using the trapezoidal rule [19]. AUC values among groups were compared using the Mann-Whitney test. Nonparametric rank-based method was used to analyze and compare the changes over time between the groups as the data is skewed and sample size is small. The analysis was done using R package NparLD. All P values were based on a two-sided hypothesis, and P<0.05 was considered statistically significant.

## Results

**Fig 1:**
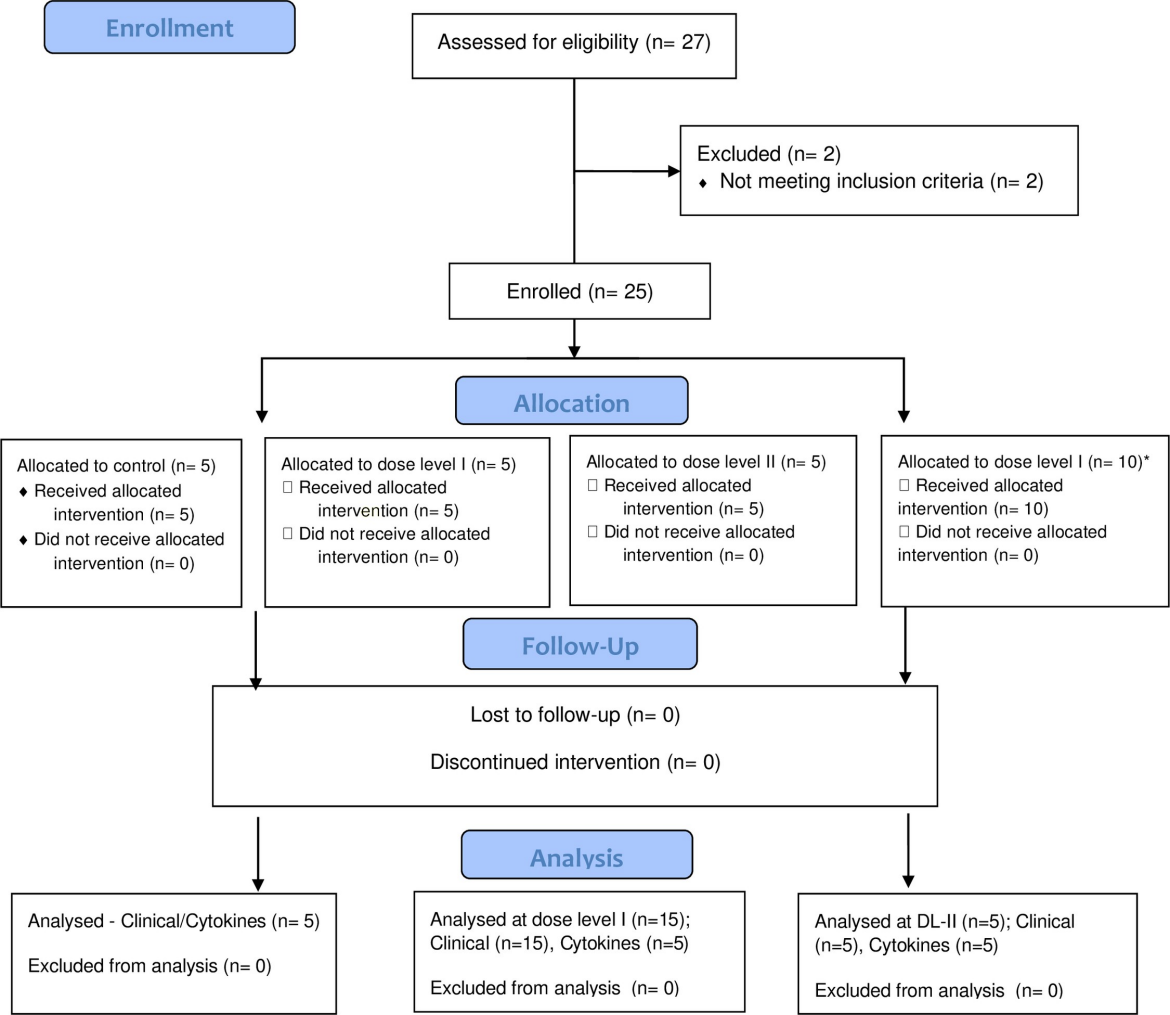
Flow chart. *Only clinical outcome was obtained from these patients. Cytokine levels were not measured.

Between February 2017 to September 2019, 25 eligible patients undergoing autologous bone marrow transplantation for multiple myeloma were enrolled in the study. The study flowchart is shown in Fig 1. For analysis of baseline parameters and toxicity grades, groups DL-I (N = 15) and DL-II (N = 5) were combined (N = 20), and were compared with the control group (N = 5). Baseline characteristics of patients in the two groups are illustrated in Table 1. Incidence of grade 3/4 oral mucositis was significantly reduced in the resveratrol-copper group compared to control group (40% vs 100%; p = 0.039) (Table 2). Incidence of opioid use was also lower in resveratrol-copper group (55% vs100%), although this difference did not reach statistical significance (Table 2). Similarly, a non-significant difference in use of Total Parenteral Nutrition (TPN) was observed (35% in resveratrol-copper groups vs 80% in control group) (Table 2). There was no significant difference in incidence and duration of grade III/IV diarrhea, nausea or vomiting. No adverse events that could be possibly or definitely attributed to resveratrol-copper were noted.

**Table 1:** Baseline characteristics of the patients.

	Control	Resveratrol- copper- 5.6mg	Resveratrol- copper- 50mg
	(n = 5)		
		(n = 15)	(N = 5)
Gender (M:F)	4:1	15:0	4:1
	(80%:20%)	(100%: 0%)	(80%, 20%)
Median age at diagnosis (IQR)	38 (33–57)	44 (40,49)	53(45,57)
Median age at transplant (IQR)	39 (37–58)	47 (42,50)	54(46,59)
Disease stage (ISS MM)			
I	3 (60%)	4 (27%)	1 (20%)
II	1 (20%)	6 (40%)	3 (60%)
III	1 (20%)	5 (33%)	1 (20%)
Disease status at transplant			
CR	2 (40%)	8 (53%)	3 (60%)
VGPR	2 (40%)	7 (47%)	2 (40%)
Progression	1 (20%)	0 (0%)	0 (0%)
Cytogenetic risk^#^			
High risk	3 (60%)	6 (40%)	2 (40%)
Standard risk	2 (40%)	9 (60%)	3 (60%)

ISS MM- International Staging System for Multiple Myeloma

CR- Complete Response; VGPR- Very Good Partial Response.

# Based on International myeloma working group cytogenetic risk stratification system.

**Table 2:** Comparison of toxicity in control and resveratrol-copper group.

Toxicity		Control	Dl-I	p value	DL-II	p value
		(n = 5)	(n = 15)		(n = 5)	
**Oral mucositis**	Incidence of grade 3–4 mucositis	5 (100%)	6 (40%)	0.038	2(40%)	0.167
	Median duration of grade 3–4 mucositis (range)	5 (2–8)	4.5 (2–6)	0.848	4.5 (3–6)	0.693
**Diarrhoea**	Incidence of grade 3–4 diarrhoea	3 (60%)	6 (40%)	0.617	3 (60%)	1.000
	Median duration of grade 3–4 diarrhoea (range)	3 (2–11)	2.5 (2–9)	0.492	1(1–3)	0.178
**Vomiting**	Incidence of grade 3–4 vomiting	0 (0%)	2 (13%)	1.000	0(0%)	1.000
	Median duration of grade 3–4 vomiting (range)	-	1 (1–1)	-	-	-
**Nausea**	Incidence of grade 3–4 nausea	1 (20%)	4 (27%)	1.000	0 (0%)	1.000
	Median duration of grade 3–4 nausea (range)	4	4.5 (2–9)	0.480	-	-
**Use of analgesics**	Incidence of use of opioid analgesics	5 (100%)	8 (53%)	0.114	3 (60%)	0.444
	Median duration of use of opioid (range)	7(5–11)	6.5(1–10)	0.374	8(4–10)	0.763
**TPN use**	Incidence of use of TPN	4 (80%)	5 (33%)	0.127	2 (40%)	0.524
	Median duration of use of TPN (range)	7(5–9)	8(7–15)	0.201	8(8–8)	0.340
**Engraftment fever**	Incidence of engraftment fever	3 (60%)	9(60%)	1.000	3(60%)	1.000

P<0.05 is statistically significant

With respect to inflammatory cytokines, resveratrol-copper caused marked modulation in terms of the AUC of serum IL—1β (p = 0.05 and p = 0.02 for DL-I and DL-II, respectively, compared to control). Also, approximately 50% reduction in exposure to serum IFN-ꝩ was observed in both DL-I and DL-II with respect to control, but the difference was not statistically significant (p = 0.057 and p = 0.068 for DL-I and DL-II, respectively). Reduction in the AUC of Salivary TNF - α (p = 0.012) and IL—1β (p = 0.009) was observed in DL-I compared to control. No significant difference in AUC was observed for any salivary cytokine among DL-II and control groups (Table 3). The trends in cytokine levels in the 3 groups is shown in S1 Fig.

**Table 3:** Cytokine exposure in serum and saliva.

	Cytokines	Control (N = 5) (pg/mL*day) Mean ± SE	DL-I (N = 5) (pg/mL*day) Mean ± SE	DL-II (N = 5) Mean ± SE	P* (DL1)	P** (DL2)
**Serum**	IL- 6	436.40 ± 72.48	339.47 ± 79.63	422.39 ± 122.85	0.394	0.924
	TNF - α	9.22 ± 7.96	45.97 ± 24.30	74.56 ± 74.56	0.072	0.700
	IL -1β	38.11 ± 9.17	79.36 ± 15.31	103.31 ± 30.47	0.050	0.022
	IL- 10	295.07 ± 138.58	66.64 ± 21.77	285.86 + 69.17	0.142	0.954
	IFN - γ	501.95 ± 114.14	246.75 ± 14.36	255.29 + 26.01	0.057	0.068
**Saliva**	IL- 6	199.88 ± 17.53	148.60 ± 25.84	648.94 ± 457.92	0.139	0.917
	TNF - α	425.83 ± 217.12	73.68 ± 15.67	358.94 + 44.60	0.012	0.678
	IL-1β	430.87 ± 257.76	60.68 ± 5.82	484.24 + 411.47	0.009	0.117
	IL- 10	803.12 ± 208.20	444.26 ± 75.50	618.53 + 93.47	0.144	0.442
	IFN - γ	590.91 ± 40.32	666.73 ± 86.37	631.54 + 72.42	0.467	0.641

P<0.05 is statistically significant

*Comparing DL-I versus Control

**Comparing DL-II versus Control

## Discussion

The impetus for the present study came from our pre-clinical findings that toxicity of chemotherapy can be prevented by a combination of the nutraceuticals resveratrol and copper with the generation of free-radicals which degrade cell-free chromatin particles [9]. In our pre-clinical study, the effective molar ratio of resveratrol to copper was found to be 1: 10^−4^ [9, 13]; the same molar ratio was maintained in the current clinical study.

The most significant finding of the present study was a reduction in grade 3/4 mucositis in resveratrol-copper patients and significant reduction in levels of certain inflammatory cytokines in serum and saliva. The present study was designed as an exploratory study, in a limited number of patients, to investigate not only the efficacy of resveratrol-copper in decreasing chemotherapy related toxicities, but also to establish the dose-response effect of resveratrol-copper on these toxicities in patients with multiple myeloma. We note that the incidence of severe mucositis, and use of TPN was high in our control group, and higher than what is usually seen in clinical practice. Because the control group was small, this could be a chance occurrence. It needs to be pointed out that, while a significant efficacy of resveratrol-copper with respect to oral mucositis was confirmed, the dose escalation clause provided in the protocol was amended. This was because, following the recruitment of first 15 patients (control, DLI and DLII), the toxicity reducing efficacy of DL-II was found not to be superior to DL-I. Consequently, 10 additional patients were recruited in DL-I (N = 15) in place of those who would have been allocated to DL-III and DL-IV.

Importantly, the reduced toxicity in DL-I was associated with reduction in the levels of proinflammatory cytokines such as TNF-α and IL-1β in saliva and serum levels of IFN-ꝩ, although the reduction in serum IFN-ꝩ was not statistically significant. Paradoxically, the serum levels of IL-1β in the resveratrol-copper group (both DL-I and DL-II) was higher compared with the control group. While a 7-fold reduction in IL-1β level was observed in saliva in DL-I, the serum levels increased by 2-fold. IL-1β is expressed in a wide variety of tissues including the lymphoid organs and the tissue macrophages of non-lymphoid organs such as liver, digestive tract and lung [20]. Thus, IL-1β modulation in saliva is likely to be more important for the prevention of mucosal injury related adverse events.

There are several agents which are being evaluated for prevention of mucosal injury following high dose chemotherapy (and / or radiotherapy)–palifermin [21], Curcumin [22] etc. Among these, palifermin is the only one which is approved by the US FDA for use in transplant setting. However, palifermin is prohibitively expensive for use in low-middle income countries like India. Pharmaco-economic analysis has shown an addition cost of approximately 5500 USD (per day of patient-controlled analgesia avoided) with palifermin in myeloma transplants [23]. Hence there is a dire need for an agent which has low cost and hence is feasible to be used in a wider population.

We did not observe any toxic side effects that could be directly attributed to free radical induced oxidative stress. This may be explained by the possibility that entry of free radicals into cells may have resulted in up-regulation of cellular antioxidant enzymes which neutralized the invading offending agents, and thereby protecting the genome from oxidative stress. Another explanation could be that R-Cu generated free radicals destroyed extracellular cell-free chromatin particles, thereby preventing their entry into cells to damage genomic DNA and cellular organelles.

Resveratrol has been extensively investigated for its anti-cancer activities. Most of these studies have been done *in vitro*, some of which have shown promising results and have shown to have anti-cancer activities. Effect of resveratrol on various cancer related pathways have been extensively reviewed [24, 25]. However, fewer studies have been conducted *in vivo*, and positive effects of resveratrol in animal models and humans have been inconsistent, possibly because of its poor bioavailability [26]. Thus, currently resveratrol is not used in routine clinical practice.

Clearly, the results of our study need to be confirmed in a randomized trial. Nonetheless, this exploratory study suggests that a combination of two relatively inexpensive and non-toxic nutraceuticals can reduce transplant related toxicity. Finally, it needs to be emphasized that the effective dose of R (DL-I) used in this study was nearly 100 times less (5.6 mg b.d. vs 500 mg b.d), and that of copper was nearly 10, 000 times less (560 ng b.d. vs 5 mg b.d.) than the recommended doses of resveratrol and copper when used as health supplements.

## Conclusions

A combination of the widely available nutraceuticals resveratrol and copper, when used in miniscule doses, can reduce transplant related toxicities in patients with multiple myeloma. This study reaffirms our pre-clinical findings that resveratrol-copper may prevent chemotherapy related toxicity by modulating the inflammatory cytokines. The findings have to be confirmed in a large randomized trial.

## Supporting information

S1 Fig Spaghetti plots of serum and saliva cytokine levels. Spaghetti plots showing trends in serum and salivary cytokine levels in dose levels 1 and 2 (DL-1 and DL-2) versus control. Only serum IFN-ꝩ showed a significant difference in trend between DL-2 and control groups (P<0.05). No significant trend was observed for any other cytokine.
(TIF)

S1 Table Details of procurement of resveratrol, copper and ELISA kits. (DOC)

S1 Appendix Raw data. (XLS)

S2 Appendix Study protocol. (DOCX)
