# Supplementary figures and images for: A novel pro-oxidant combination of resveratrol and copper reduces transplant related toxicities in patients receiving high dose melphalan for multiple myeloma (RESCU 001)
Source: PLoS One. 2022 Feb 4;17(2):e0262212. doi: 10.1371/journal.pone.0262212 (PMC8815866; doi:10.1371/journal.pone.0262212)

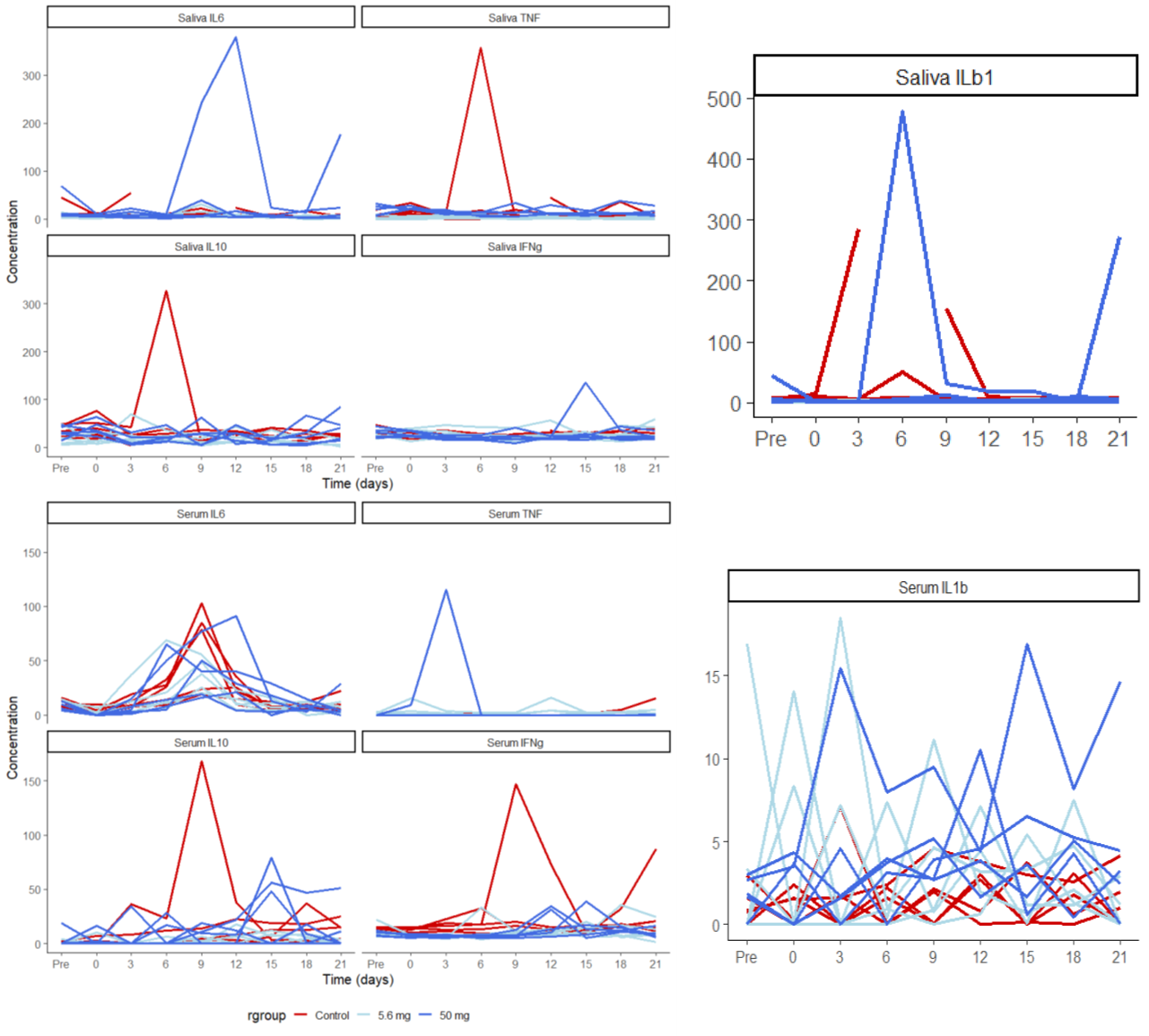

Supplement: S1 Fig — Spaghetti plots showing trends in serum and salivary cytokine levels in dose levels 1 and 2 (DL-1 and DL-2) versus control. Only serum IFN-ꝩ showed a significant difference in trend between DL-2 and control groups (P<0.05). No significant trend was observed for any other cytokine. (TIF) [file pone.0262212.s001.tif]
