# Supplementary material for: A novel pro-oxidant combination of resveratrol and copper reduces transplant related toxicities in patients receiving high dose melphalan for multiple myeloma (RESCU 001)
Source: PLoS One. 2022 Feb 4;17(2):e0262212. doi: 10.1371/journal.pone.0262212 (PMC8815866; doi:10.1371/journal.pone.0262212)
Supplement: S1 Table — (DOC) [file pone.0262212.s002.doc]

**S1 Table.** Details of procurement of Resveratrol, Copper and ELISA kits

| **Sr. No.** | **Chemicals** | **Trade name** | **Source** |
| --- | --- | --- | --- |
| 1 | Resveratrol | TransMaxTR | Biotivia LLC, USA |
| 2 | Copper | Chelated Copper | J R Carlson Laboratories Inc. USA |
|  | | | |
| **Sr. No.** | **Kits** | **Catalogue Number** | **Source** |
| 1 | IL - 6 | 555220 | BD OptEIA™, USA |
| 2 | TNF - α | 555212 | BD OptEIA™, USA |
| 3 | IL - 1β | 557953 | BD OptEIA™, USA |
| 4 | IL - 10 | 555157 | BD OptEIA™, USA |
| 5 | IFN - γ | 555142 | BD OptEIA™, USA |
