# Supplementary material for: A novel pro-oxidant combination of resveratrol and copper reduces transplant related toxicities in patients receiving high dose melphalan for multiple myeloma (RESCU 001)
Source: PLoS One. 2022 Feb 4;17(2):e0262212. doi: 10.1371/journal.pone.0262212 (PMC8815866; doi:10.1371/journal.pone.0262212)
Supplement: S2 Appendix — (DOCX) [file pone.0262212.s004.docx]

**S2 Appendix. Study Protocol**

**A study to assess the effect of Resveratrol-Copper (R-Cu) on inflammatory cytokines in multiple myeloma patients undergoing autologous peripheral blood stem-cell transplantation**

**Clinical Trial Registry India (CTRI) Registration No.: CTRI/2018/02/011905**

**Study protocol version 4 dated August 2018**

**Principal Investigator: Dr. Indraneel Mittra**

**Co- Principal Investigators: Dr. Sudeep Gupta, Dr Navin Khattry, Dr. Vani Parmar, Dr. Vikram Gota**

**Resveratrol-copper –**

**Autologous Transplant Study Protocol**

1. **Title:**

A study to assess the effect of Resveratrol-Copper (R-Cu) on inflammatory cytokines in multiple myeloma patients undergoing autologous peripheral blood stem-cell transplantation.

1. **Hypothesis:**

Oral resveratrol plus copper (R-Cu) will reduce the levels and effects of inflammatory cytokines involved in chemotherapy related toxicities such as mucositis, diarrhea and neutropenia caused by high dose chemotherapy in autologous transplant setting.

1. **Aims:**
2. To study the effect of oral R-Cu on circulating levels of inflammatory cytokines during the initial 3 weeks after stem cell infusion (counting the day of 1^st^ stem cell infusion as day 0) in patients undergoing autologous stem cell transplant for multiple myeloma.
3. To evaluate the effect of R-Cu on post-transplant toxicities, such as mucositis, diarrhea and neutropenia.

1. **Primary Objective:**

To determine the plasma and saliva levels of 6 selected inflammatory cytokines at pre-defined time-points following administration of 4-dose-levels of R-Cu in patients receiving autologous transplant for multiple myeloma. Both Resveratrol and copper are available as over-the-counter health supplements.

1. **Secondary Objectives:**
   1. To quantify toxicities by NCI Common Toxicity Criteria version 4.03 in myeloma patients undergoing autologous stem cell transplant who have been administered 4-dose-levels of R-Cu.
   2. To quantify serum chromatin levels at predefined time points following administration of 4-dose-levels of R-Cu in these patients.
2. **Exploratory Objectives:**
   1. To estimate total leukocyte, absolute neutrophil and platelet counts at predefined time points following administration of 4-dose-levels of R-Cu.
   2. To quantify DNA-damage, apoptosis and activation of inflammatory cytokines in PBMCs following administration of 4-dose-levels of R-Cu.
   3. To quantify serum lipid profiles at predefined time points following administration of 4-dose-levels of R-Cu.
   4. To evaluate the steady-state PK parameters for resveratrol and copper on Day 7 in patients receiving autologous stem cell transplant at 4-dose levels of R-Cu.
3. **Background :**

Resveratrol is a poly-phenolic stilbenoid naturally present in the skin of red grapes and other fruits and berries, peanuts and also in the roots of Japanese knotweed.**^1^** Resveratrol has been shown to have multiple health benefits that include life extension, cancer prevention, cardio-protection, neuro-protection and anti-diabetic, anti-inflammatory and anti-viral activities.**^2 – 8^** These actions are thought to be mediated through its intrinsic anti-oxidant properties and the ability of Resveratrol to activate SIRT1.**^9 – 11^** However most of the positive effects exhibited by Resveratrol could not be replicated in clinical trials possibly because of its low bio-availability.**^12, 13^**

Copper (Cu) is an essential micronutrient, and because of its role as a metal co-factor, has the ability to generate reactive oxygen species (ROS), *viz*., O_2_^-.^ and **^•^**HO radicals.**^14^** Fukuhara and Miyata were first to show that Resveratrol can act as a pro-oxidant in the presence of Cu and cause oxidative DNA cleavage in a pBR322 plasmid assay.**^15^** Resveratrol forms a complex with Cu (II), leading to its reduction to Cu (I) with concomitant production of ROS which is responsible for DNA scission.**^16^** R-Cu was shown to be active in biological systems as evidenced by its ability to inactivate bacteriophages**^8^** and to cause fragmentation of DNA of human lymphocytes *in vitro*.**^17^** These findings have led to the proposal that R-Cu could be used in the prevention and treatment of cancer.**^17, 18^**

The DNA cleaving activity of R-Cu was reported with respect to cleavage of plasmid DNA. **^15^** We have recently reported that R-Cu can also cleave genomic DNA and RNA.**^19^** We further reported the surprising observation of a paradoxical synergistic effect between Resveratrol and Cu whereby genomic DNA cleaving / degrading activity of R-Cu increased progressively as the ratio of Resveratrol to Cu was increased i.e., the concentration of Cu was successively reduced with respect to a fixed concentration Resveratrol. Whereas cleavage of genomic DNA occurred at low molar ratios of Resveratrol to Cu, at higher ratios, complete degradation of DNA was achieved. By further increasing the ratio, whereby the concentration of Cu was reduced to very low levels, the DNA degrading activity of R-Cu was lost. This paradoxical synergistic effect is also seen with respect to eukaryotic RNA.

**Regulatory approval for use of resveratrol and copper in India**:

Resveratrol is listed as a nutraceutical as per the notification issued by the Food Safety and Standards Authority of India dated 23rd July, 2015. (http://www.fssai.gov.in/Portals/0/Pdf/Draft_Regulation_on_Nutraceuticals_WTO_23_07_2015.pdf).

In the same notification, a nutraceutical is defined as “a naturally occurring chemical compound having a physiological benefit or provide protection against chronic disease, isolated and purified from food or non-food source and may be prepared and marketed in the food-format of granules, powder, tablet, capsule, liquid or gel and may be packed in sachet, ampoule, bottle, etc and to be taken as measured unit quantities.” It is also available as a health supplement over-the-counter in many countries.

Copper salts are also listed in Schedule II of the notification under food for special medical purposes (FSMP) wherein up to 500μg of copper can be used per 100 kcal of food consumed per day. For example an average size adult human who consumes 2000kcal of food per day is allowed consumption of up to 10 mg of copper per day.

**Clinical studies using resveratrol**:

Resveratrol has been extensively studied in clinical trials in humans in a range of therapeutic doses in healthy human volunteers as well as in patients with diabetes, obesity and cancer. The studies are summarized in Table no.1 below. Overall the data indicates that there are no major safety concerns with doses ranging up to 5000 mg/day. In some studies, higher doses caused mild gastrointestinal intolerance in a small fraction of patients.

In one study in myeloma patients, there were concerns about renal toxicity (Popat et al., Table 1); however, all patients of myeloma were at risk of this complication, the dose of resveratrol (5000 mg / day) was 5 times higher than our highest proposed dose and the formulation of resveratrol was a more orally bioavailable micronized version.

Given the above observations, it is reasonable to state that the dose levels prescribed in our study are highly unlikely to result in major toxicities.

Table 1:

| AUTHOR | PARTICIPANTS | FORM AND DOSE OF RESVERATROL | OUTCOME |
| --- | --- | --- | --- |
| Almeida  et al. 2009 | Healthy men (20)  and women (20) | Dose / day = 150mg, 300mg, 600mg or 900mg | Resveratrol was well-tolerated, but with some mild adverse events reported |
| Boocock et al. 2007 | Healthy men (18)  and women (22) | Dose / day = 500mg, 1000mg, 2500mg or 5000mg | Consumption of resveratrol did not cause serious adverse events. |
| Brown et al. 2010 | Healthy men (22) and women (18) | Dose / day = 500mg, 1000mg, 2500mg or 5000mg | 2500mg and 5000mg caused mild to moderate gastrointestinal symptoms *“including nausea, flatulence, abdominal discomfort, and diarrhea. Most of these events were mild (severity grade 1, NCI CTCAE v.4.0), although four participants on the 2.5 and 5.0 g doses presented with nausea and/or diarrhea of moderate severity (grade 2).”* |
| Burkon et al. 2008 | Healthy males (9) | Dose / day = 85.5mg | No adverse events were reported in this study. |
| La Porte et al. 2010 | Healthy men (3) and women (5) | Dose / day = 4000mg | Resveratrol was well tolerated, “*although diarrhea was frequently observed (6 out of 8 subjects*).” |
| Nunes et al. 2009 | Healthy young men (6) and healthy young women (6) and elderly men (6) and elderly women (6) | Dose / day = 600mg | Resveratrol was well tolerated by young and elderly subjects.  “*Reported adverse events were nonspecific and mild in severity. No clinically relevant abnormalities were found in the 12-lead ECG parameters or clinical laboratory safety tests*.” |
| Patel et al. 2010 | Colon cancer patients (20) | Dose / day = 500mg or 1000mg | *“Resveratrol was found to be well tolerated.”* |
| Ortuno et al. 2010 | Healthy men (11) | Dose / day = 980µg | No adverse events were reported in this study. |
| Walle et al. 2004 | Healthy men (3) and healthy women (3) | Dose / day = 25mg | No adverse events were reported in this study. |
| Goldberg et al. 2003 | Healthy men (12) | Dose / day = 25mg | No adverse events were reported in this study. |
| Ghanim et al. 2010 | Healthy adults (20) | Dose / day = 40mg | No adverse events were reported in this study. |
| Ghanim et al. 2011 | Healthy men (4) and women(6) | Dose / day = 175mg | No adverse events were reported in this study. |
| Kennedy et al. 2010 | Young healthy men (4); women (20) | Dose / day = 250mg or 500mg | No adverse events were reported in this study. |
| Wong et al. 2010 | Overweight/ obese men (14);  postmenopausal women (5) with borderline hypertension | Dose / day = 30mg, 90mg or 270mg | No adverse events were reported in this study. |
| Nguyen et al. 2009 | Colorectal cancer patients (8) | Dose / day = 20mg or 80mg | *“No patients experienced any toxicities while enrolled on the clinical trial, regardless of whether they received resveratrol tablets or the freeze-dried GP.”* |
| Chow et al. 2010 | Healthy men (11) and women (31) | Dose / day = 1000mg | *“Four weeks of daily administration of pharmacologic doses of resveratrol was well tolerated in healthy participants. All reported adverse events were Common Toxicity Criteria grade 1 or 2, and many were very mild and transient. One participant withdrew from study participation after the first dose of resveratrol due to diarrhea. One postmenopausal woman (body mass index, 36.9 kg/m^2^) experienced new onset, persistent perimenopausal symptoms (hot flashes, insomnia), which required a 50% dose reduction. Four weeks of resveratrol dosing did not result in any clinically significant changes in blood chemistry and hematology measurements (data not shown).”*  However, the study does advice: *“pharmacologic doses of resveratrol could potentially lead to increased adverse drug reactions or altered drug efficacy due to inhibition or induction of certain CYPs. Further clinical development of resveratrol for cancer prevention should consider evaluation of lower doses of resveratrol to minimize adverse metabolic drug interactions.”* |
| Elliot et al. 2009 | Healthy normal volunteers and  type 2 diabetes patients | Dose / day = 2500mg or 5000mg | *“Our results demonstrate that SRT501 was safe and well tolerated in multiple preclinical toxicity studies and in clinical trials involving healthy normal volunteers and type 2 diabetes patients.”* |
| Brasnyo et al. 2011 | Type 2 diabetic men (19) | Dose / day = 10mg | No adverse events were reported in this study. |
| Timmers et al. 2011 | Healthy obese men (11) | Dose / day = 150mg | *“No clinical adverse events were reported during the resveratrol supplementation.Electrocardiograms (ECGs) were inconspicuous and did not raise clinical concern in any subject.”* |
| Crandall et al. 2012 | Older men (3) and women (7) with impaired glucose tolerance | Dose / day = 1000mg, 1500mg or 2000mg | *“All doses of resveratrol were well tolerated, and there were no serious adverse events or changes in laboratory safety parameters, including serum creatinine, liver enzymes, complete blood cell count, or urinalysis. Specifically, there were no instances of transaminase increases above the normal range, and all subjects completed the protocol. Among adverse events considered “possibly” related to study treatment, one subject reported mild diarrhea and two women reported increased “hot flashes.””* |
| Popat et al. 2013 | Patients with relapsed and or refractory multiple myeloma (24) | Dose / day = 5000mg | *The predominant study finding was unexpected renal toxicity, with five SAEs of renal failure leading to early study termination. The most commonly reported adverse events were: nausea (79%), diarrhoea (71%), vomiting (54%), fatigue (46%) and anaemia (38%). 54% of patients reported _grade 3 AEs; most commonly 21% haematological (anaemia and thrombocytopenia), 21% renal failure, 13% nausea and 13% infections. 50% had a serious AE (SAE) and two deaths occurred on study (one possibly treatment related; one due to PD).*  But the study also reports that:  *“Two hundred and thirty seven patients in seven studies had previous SRT501 treatment, predominantly healthy volunteers (n = 92), but also type two diabetes (n = 136) and mitochondrial encephalomyopathy, lactic acidosis, and stroke-like episodes (MELAS) syndrome. The phase 2 dose (5 g) was safely assessed in these studies (Sirtris Pharmaceuticals, Inc, 2009). Furthermore, a Phase 1 study for metastatic colorectal cancer patients (Howells et al, 2011), did not report nephrotoxicity. As SRT501 is extensively metabolized, renal failure seemed specific to MM patients. Renal impairment can occur in up to 50% of MM patients due to multiple causes, hence all are at risk.”* |

**Clinical studies using copper**:

The World Health Organization has set an upper value of copper concentration considered to be safe in drinking water at 2.0 mg Cu/L and the US Food and Nutrition Board has set the tolerable upper intake level (UL) for copper at 10 mg/day from food and supplements.

Copper supplementation has been studied in clinical trials in humans in a range of doses in healthy volunteers and in patients diagnosed with Alzheimer’s disease. The studies are summarized in Table 2 below.
In one study in healthy volunteers, copper at a dose of 7.8 mg/day altered the index of oxidant stress and affected several indices of immune function. Of note, the dose of copper (7.8 mg/day) in this study was 1.6 times higher than our highest proposed dose.
Data from a clinical study involving patients diagnosed with Alzheimer’s disease indicated that there were no major safety concerns with doses ranging up to 8mg / day.

Given the above observations, it is reasonable to assume that the dose levels prescribed in our study are highly unlikely to result in major toxicities.

Table 2:

| AUTHOR | PARTICIPANTS | FORM AND DOSE OF COPPER | OUTCOME |
| --- | --- | --- | --- |
| Fitzgerald DJ | NA | NA | *“The principal health-based guideline values (for the safe ingestion of copper in drinking water) have been set by the US Environmental Protection Agency (1.3 mg Cu/L) and the World Health Organization (2.0 mg Cu/L).”* |
| Food and Nutrition Board, Institute of Medicine, USA. | NA | NA | *“The US Food and Nutrition Board set the tolerable upper intake level (UL) for copper at 10 mg/day from food and supplements.”* |
| Turnlund et al. 2004 | Healthy men (9) | Dose / day = 1.6mg for 18 days  7mg for 129 days  7.8mg for 18 days | *“Under highly controlled conditions, long-term high copper intake results in increases in some indexes of copper status, alters an index of oxidant stress, and affects several indexes of immune function. The physiologic implications of these changes are unknown.”* |
| Turnlund et al. 2005 | Healthy men (9) | Dose / day = 1.6mg for 18 days  7mg for 129 days  7.8mg for 18 days | *“Several indexes of immune function changed. Neutrophils decreased, lymphocytes increased, and interleukin 2 receptor decreased. Serum antibody titers after immunization were much lower in subjects who received supplements than in control subjects who did not receive supplements.”* |
| Kessler et al. 2008 | Patients with a diagnosis of probable Alzheimer’s disease (68) | Dose / day = 8mg | *“None of the serious adverse events [hospitalizations due to intracranial hemorrhage (1 patient), diagnosis of non-Hodgkin lymphoma (1 patient) and suspected cerebral infarction (1 patient)] leading to discontinuation of the study was considered to be attributable to Cu supplementation.*  *The treatment was well-tolerated. The present clinical trial demonstrates that (1) long-term oral intake of 8 mg Cu can be excluded as a risk factor for AD. (2) Long-term oral intake of Cu is well-tolerated by AD patients. (3) Cu intake has no effect on the progression of AD.”* |

1. **Basis for correlating inflammatory cytokines with chemo-toxicity:**

Inflammatory cytokines play an important role in chemotherapy related toxicities such as oral mucositis and diarrhea.**^47^** In particular the following cytokines have been implicated in chemotherapy toxicity: NFκB, TNFα and IL-1β. **^47^**

The ACTREC Curcumin Study:

Inflammation in relation to chemotherapy-related toxicities has also been investigated in a study conducted at ACTREC in bone marrow transplant patients. In this the efficacy of curcumin in reducing toxicity of high dose melphalan (200 mg/m^2^) in patients of multiple myeloma undergoing autologous transplant was evaluated. Salivary and serum cytokines were measured at predetermined time points in a cohort of patients (n=10) who did not receive curcumin and the levels of cytokines both in serum and saliva of this cohort were compared to a subsequent cohort (n=27) of patients who received curcumin at a dose of 400 mg BD from 2 days prior to till 28 days post transplant. The serum and salivary cytokines measured were IL-1,IL-6, IL-8, IL-17,TNF alpha, TGF beta, PGE2 and interferon gamma. The median levels of various cytokines in serum and saliva over 28 days post transplant were compared in the 2 groups as shown in Annexure 1. For example, it can be seen in Figure 1 that the median serum levels of interferon gamma **(IFN-γ)** were reduced in the curcumin group on day+13 and day+28 (P=0.067 and 0.040 respectively) compared to the control group.

P value

Day+13=0.067

Day+28=0.040

Figure-1:- Median serum INF-γ levels at each time point between the two groups.

**Serum IFN-γ**

1. **Preclinical studies implicating circulating chromatin in chemotherapy induced inflammation and DNA damage:**
2. **Chemotherapy induces elevation of blood chromatin levels.**

6-8 week old female C57/Bl6 mice weighing ~20g were divided into 3 groups (10 mice each). Control animals received 100µl saline alone. Animals in the 2 test groups were given 10 mg/kg Adriamycin i.p. as a single injection in 100µl of saline. One group received in addition to Adriamycin, Resveratrol and Cu by oral gavage in the dose levels described above. The first dose of R-Cu was administered 4hr prior to Adriamycin injection and 12 hourly thereafter. Blood was collected by orbital puncture 36hr after Adriamycin injection. Chromatin levels were estimated in duplicate using Cell Death Detection ELISA^plus^ kit (Roche Diagnostics GmbH, Germany).


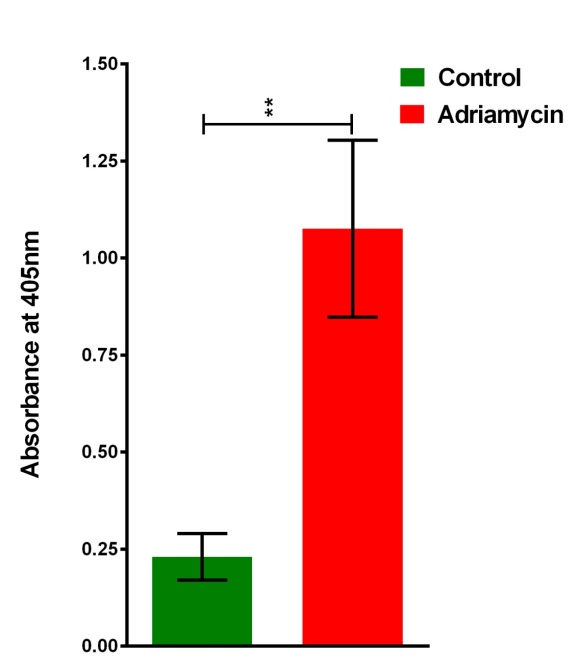


Figure 2: The histogram shows that there was nearly 5-fold increase in chromatin level following Adriamycin injection. ** p<0.01.

1. **Circulating chromatin causes systemic inflammation and DNA damage**

We have reported that when chromatin fragments (Cfs) isolated from human subjects are injected intravenously into mice, they integrate into genomes of their vital organs and damage their DNA and activate inflammatory cytokines (Mittra et al., 2015). Representative images are given below.

**Inflammation**:

Figure 3: Activation of inflammatory cytokines NFκB, IL-6, TNFα and IFNγ in vital organs of animals injected with 100ng DNA equivalent of chromatin isolated from cancer patients. PCfs = purified chromatin.

**
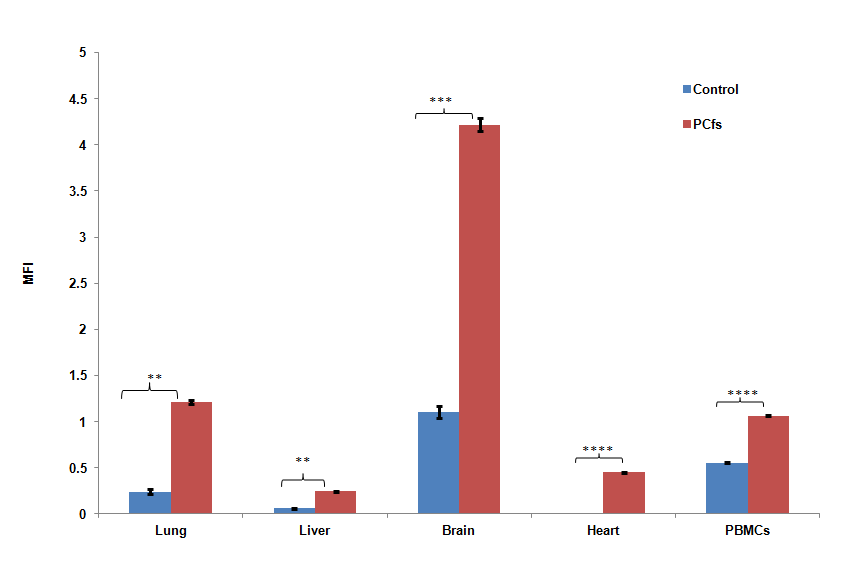
NFκB**

Figure 4: Quantification of inflammatory cytokine (NFκB) activation in vital organs of animals injected with 100ng DNA equivalent of chromatin isolated from cancer patients. PCfs = purified chromatin. ** p<0.01, *** p<0.001, **** p<0.0001

**DNA damage**:


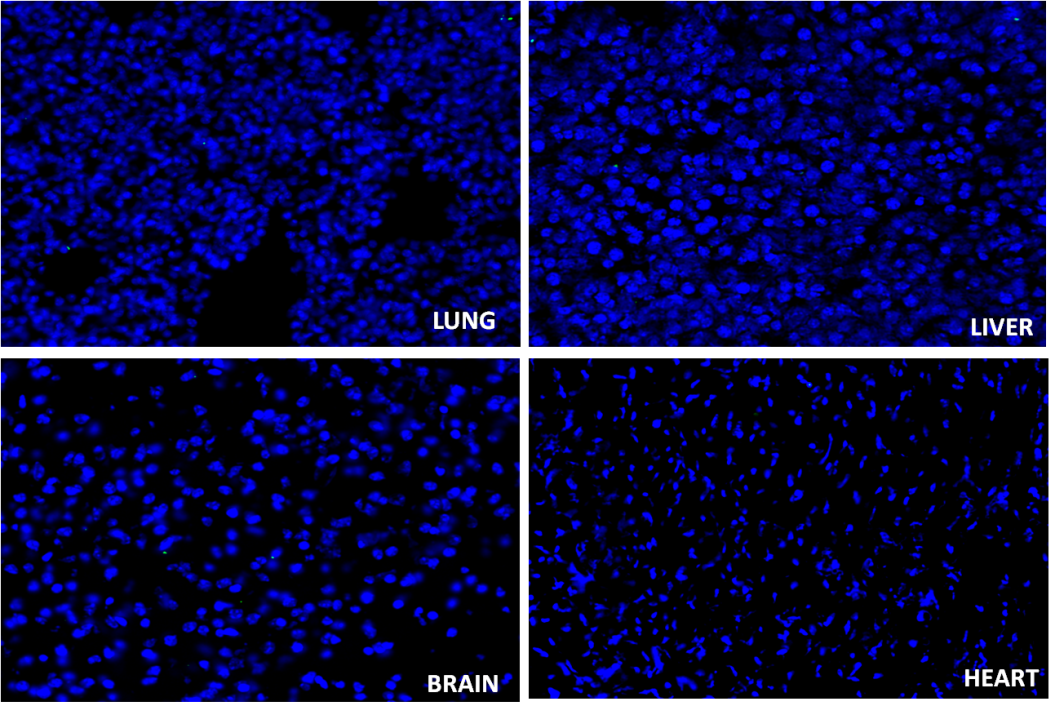
**Control animals**


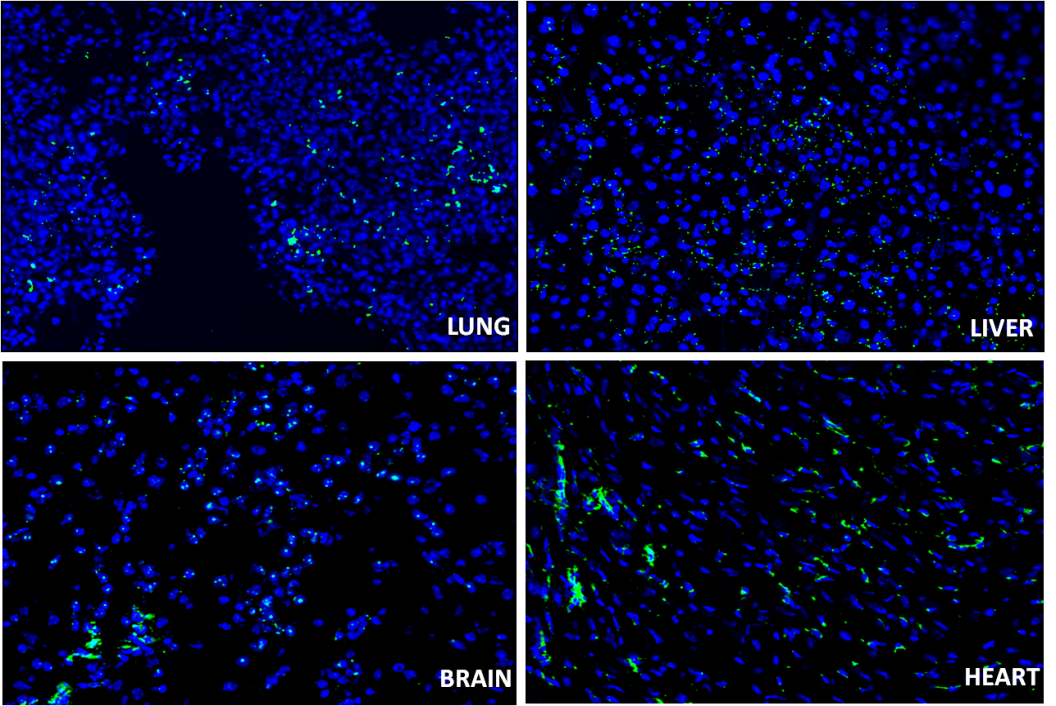
**Chromatin injected animals**

Figure 5: Activation of H2AX, an indicator of DNA double strand breaks in vital organs of animals injected with 100ng DNA equivalent of chromatin isolated from cancer patients.

**γ
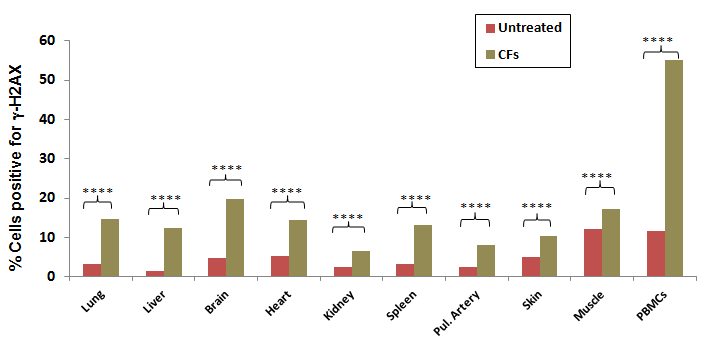
-H2AX**

Figure 6: Quantification of H2AX, an indicator of DNA double strand breaks in vital organs of animals injected with 100ng DNA equivalent of chromatin isolated from cancer patients. Cfs = purified chromatin. * p<0.05, **** p<0.0001, NS = not significant

1. **Chromatin degrading agent resveratrol-copper prevents chemotherapy induced inflammation, DNA damage and apoptosis**

Based on these findings, we hypothesized that much of the toxicity of chemotherapy may not be due to the drug itself, but rather due to chromatin fragments released from initial round of cell-death induced by chemotherapy followed by a cascading effect of chromatin-induced DNA damage, apoptosis and inflammation.

To test this hypothesis, we estimated the following biomarkers of Adriamycin toxicity following a single injection of the drug: 1) NFκB (marker of inflammation); 2) IL-6 (marker of inflammation); 3) γ-H2AX (marker of dsDNA breaks); and 4) active Caspase-3 (marker of apoptosis). NFκB and IL-6 were estimated 72hr after Adriamycin treatment (10mg / kg) while γ-H2AX and active Caspase-3 were estimated 24hr following i.p. Adriamycin treatment. The various biomarkers were estimated in the following organs / tissues: lung, liver, heart, brain, ovary, skin and small intestine. R-Cu treatment was started 4hr prior to Adriamycin and every 12hr thereafter. The above biomarkers were estimated by indirect immnuoflourescence. There were 5 animals in each group. One thousand cells were analyzed for each organ and the mean fluorescence intensity of each biomarker in each group was estimated and compared by student’s t-test.

In addition to R-Cu, we also used two other chromatin degrading / neutralizing agents namely, DNase I and anti-histone antibody complexed nanoparticles (CNPs).


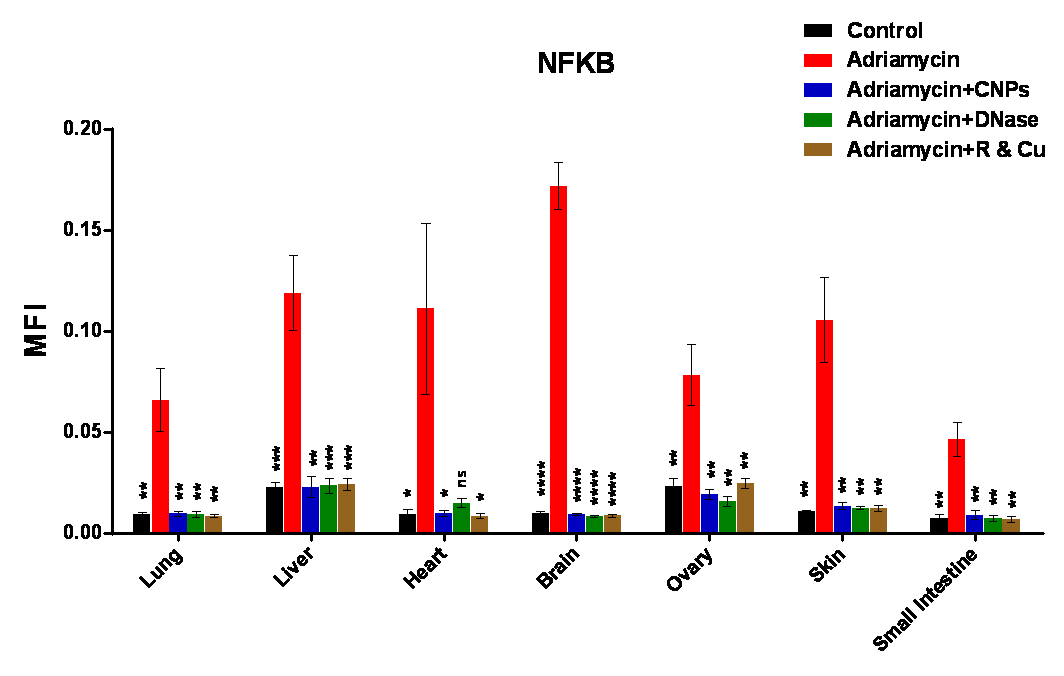


**A**


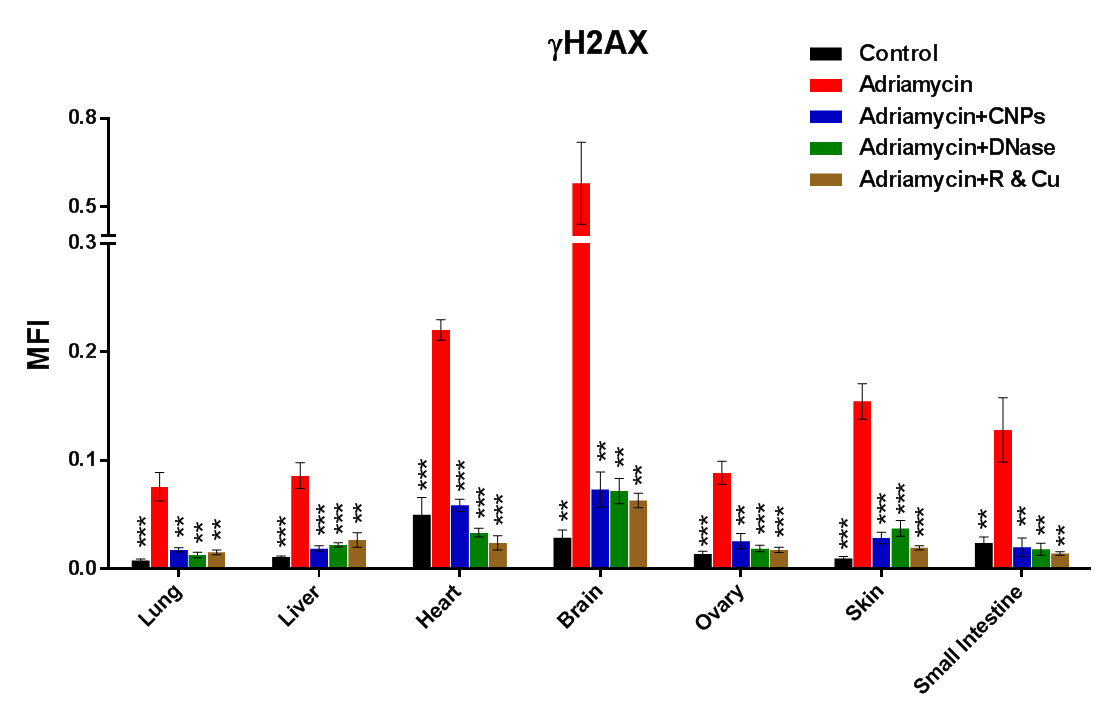

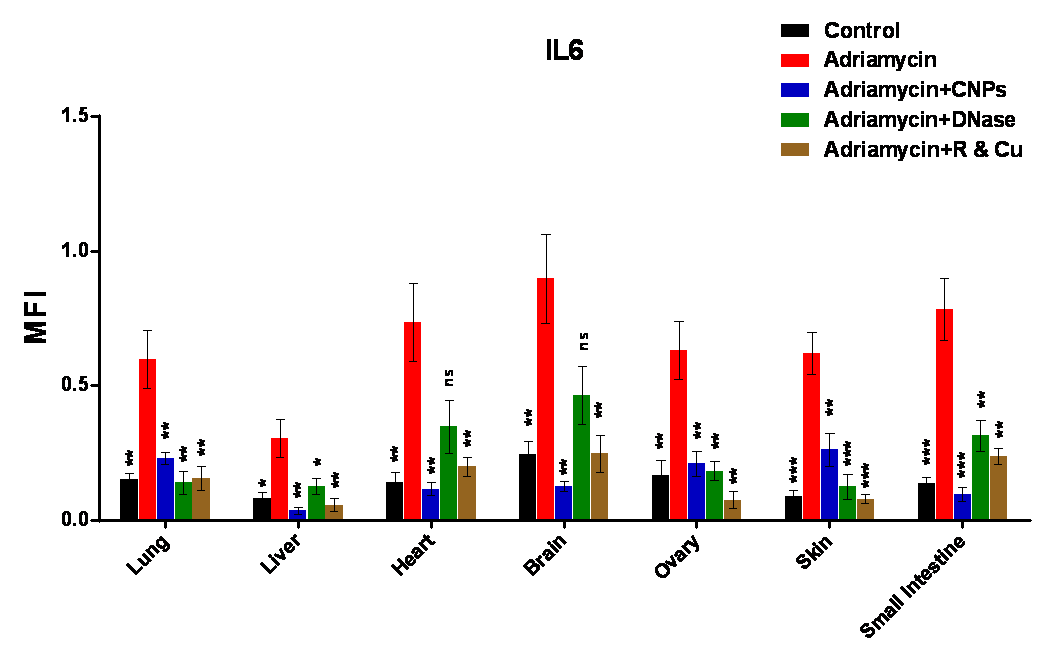


**C**

**B**

**B**


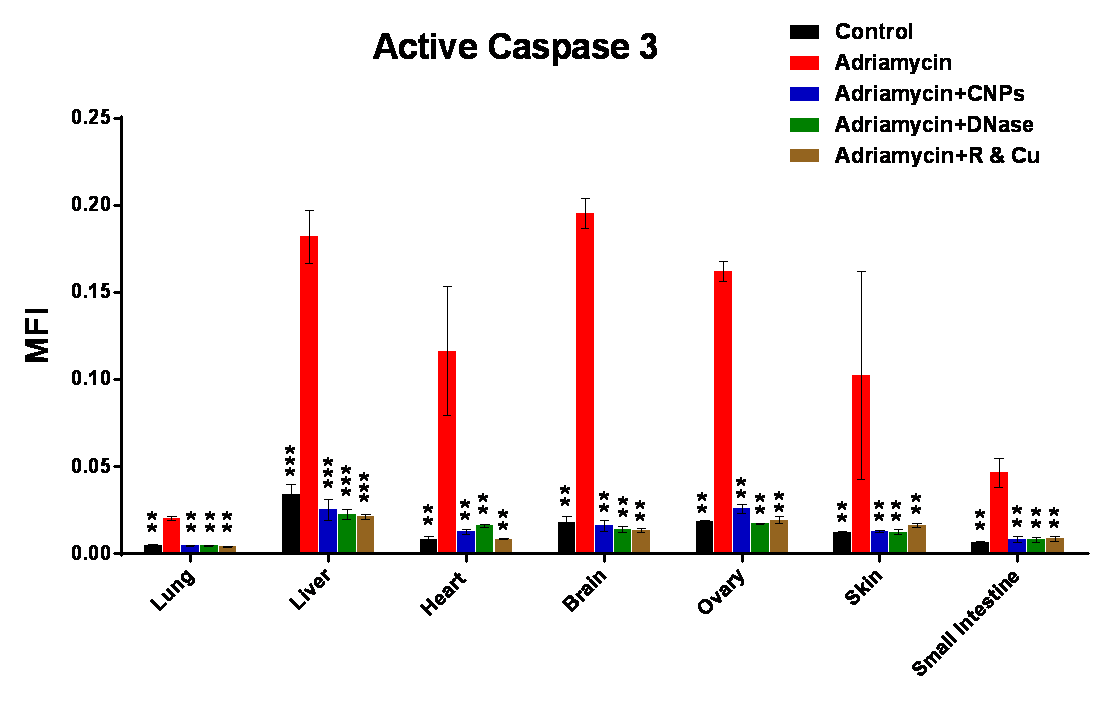


**D**

**Figures 7A – D: The above histograms show that all 3 chromatin degrading / neutralizing agents prevented the rise of all 4 biomarkers to near-baseline levels in all tissues tested.**

This data validate our hypothesis that toxicity of chemotherapy is largely, if not entirely, due to chromatin fragments arising from the initial round of cell death induced by a chemotherapeutic drug.

1. **Chromatin degrading agent resveratrol-copper prevents chemotherapy induced neutropenia**

We hypothesized that neutropenia following chemotherapy is not due to the drug itself, but rather due to chromatin fragments released from initial round of leukocyte cell-death induced by chemotherapy followed by a cascading effect of chromatin-induced leukocyte cell-death causing further rounds of leukocyte cell-death. This might explain why neutropenia following chemotherapy lasts for nearly 10 days when the half-life of most chemotherapy drugs are a few minutes to an hour.


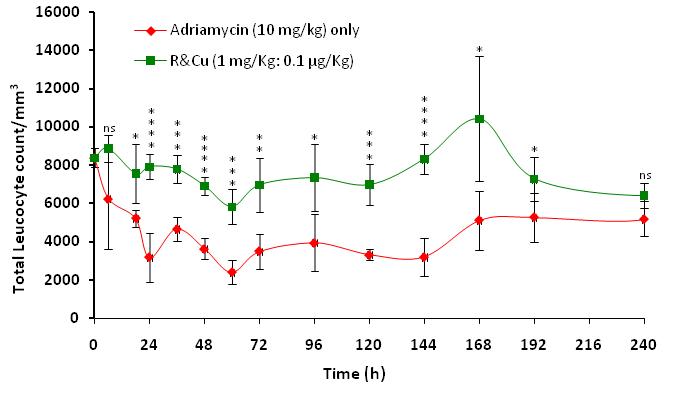
Mice were divided into 2 groups of 10 animals each. The control group received a single i.p. injection of Adriamycin (10mg/ kg). Blood was collected from 5 mice from each group on alternate days by orbital puncture for total leukocyte count at each time-point as shown in the figure. Animals in the test group received in addition to Adriamycin, R-Cu by oral gavage starting 4hr prior to chemotherapy and every 12hr thereafter.

Figure 8: The graph above shows that neutropenia induced by Adriamycin could be significantly prevented throughout the 10 days of observation. This data validates our hypothesis that neutropenia following chemotherapy is largely due to chromatin fragments arising from the initial round of cell death induced by a chemotherapeutic drug.

1. **Chromatin degrading agent resveratrol-copper prevents chemotherapy induced lethality**

Based on the above studies, we tested if R-Cu could prevent death in mice following a lethal dose of Adriamycin. In addition to R-Cu, we also used two other chromatin degrading / neutralizing agents namely, DNase I and anti-histone antibody complexed nanoparticles (CNPs).


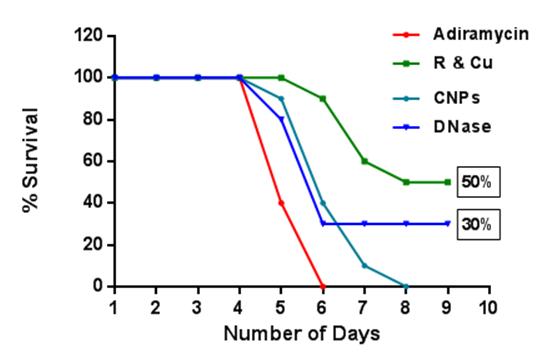
Mice were divided into 4 groups of 10 animals each. The control group received a single i.p. injection of lethal dose of Adriamycin (20mg/ kg) while the other 3 groups received, in addition to Adriamycin, R-Cu; CNPs and DNase I. The animals were observed for lethality.

Figure 9: The above graph shows that all animals receiving Adriamycin alone died by Day 6. On the other hand, R-Cu prevented 50% of the deaths, while DNase I prevented 30% of the deaths. All animals in the CNPs group died; nonetheless the life of the animals were prolonged by ~2 days.

This data shows that R-Cu is capable of preventing lethality from Adriamycin and indicates that mortality from chemotherapy is largely due to cell death caused by chromatin fragments.

1. **Preparation of Resveratrol and Cu used in pre-clinical studies**:

Exploratory studies in mice *in vivo* have confirmed the *in vitro* results that R-Cu remains active in degrading circulating chromatin even when the molar concentration of Cu with respect to Resveratrol is reduced to as low as 1: 10^-4^ or 1: 10^-6^. Based on these exploratory experiments, we have chosen the standard *in vivo* dose of R-Cu as 1: 10^-4^ (weight for weight) for all our *in vivo* work in mice. We used Trans-Resveratrol (TransMax^TR^) and copper (Chelated Copper) that are available for human use for our animal experiments. The calculated dose that we have routinely used in our animal experiments are:

**Resveratrol 1mg / kg i.e., 20 µg / 20g mouse and**

**Cu 100 ng / kg i.e., 2 ng/ 20 g mouse**.

**Both R and Cu are administered as oral gavage in 50µl of saline one after the other** **without mixing.**

Calculations for Resveratrol**:** 1 capsule containing 500 mg of water-soluble Resveratrol is dissolved slowly in 125 mL of distilled water with uniform mixing under constant grinding in pestle and mortar. This makes a stock solution of 4 mg / mL. This stock solution is further diluted 1:10 and used as working solution (0.4 mg/mL).

**50µl of this solution containing 20µg of Resveratrol is administered into a 20g mouse**.

Calculations for Chelated Copper: 1 tablet containing 5mg of chelated copper is dissolved in 100mL of distilled water to make a stock solution (A) containing 50µg / 1 ml. The next stock solution (40µg / ml) (B) is prepared by adding 200µl of distilled water to 800µl of stock solution (A). The final working solution (0.04µg / mL) (C) is prepared by a 1:1000 dilution of the stock solution (B).

**50µl of this solution (C) containing 2ng of Cu is administered into a 20g mouse**.

1. **Study Methodology:**
   1. **Study Design**

This is a prospective, single centre, pilot, pharmacodynamic and pharmacokinetic study to evaluate the effects of 4-dose-levels of R-Cu on biomarkers of inflammation and on immediate post-treatment toxicity in consecutive, eligible and consenting patients undergoing autologous peripheral blood stem-cell transplant for multiple myeloma.

- 1. **Study population**

Target populations are as follows: patients undergoing autologous hematopoietic stem cell transplantation for multiple myeloma at Tata Memorial Centre.

Inclusion Criteria-

1. Male or female patients 18 years and above.
2. Patients who have given written informed consent
3. Patients with performance status of 0,1 or 2 (ECOG scale)
4. Patients of multiple myeloma receiving melphalan**-** 200 mg/m^2^ (MEL-200 mg/m^2^)
5. Patients who have creatinine clearance > 50 ml/min
6. Patients with serum bilirubin levels < 2mg/dl and serum liver enzymes (ALT or AST or both) less than 5 times the upper limit of normal value.

Exclusion Criteria-

1. Patients who are on NSAIDs , aspirin ,antioxidants or systemic steroids for more than 3 months and the last dose taken within the last one week.
2. Patients being treated for active infection at the time of starting high dose chemotherapy
   1. **Study intervention**
      1. **Rationale for proposed human dose combinations of R and Cu:**

The animal experiments described above were done using Resveratrol at 1 mg/kg, i.e., 20 µg/20g mouse and Cu at 100 ng/kg i.e., 2 ng/20 g mouse.

To determine the lowest human doses of resveratrol and copper, we used the dose translation formula for converting mouse dose to human dose (Reagan-Shaw S *et al*., 2008).

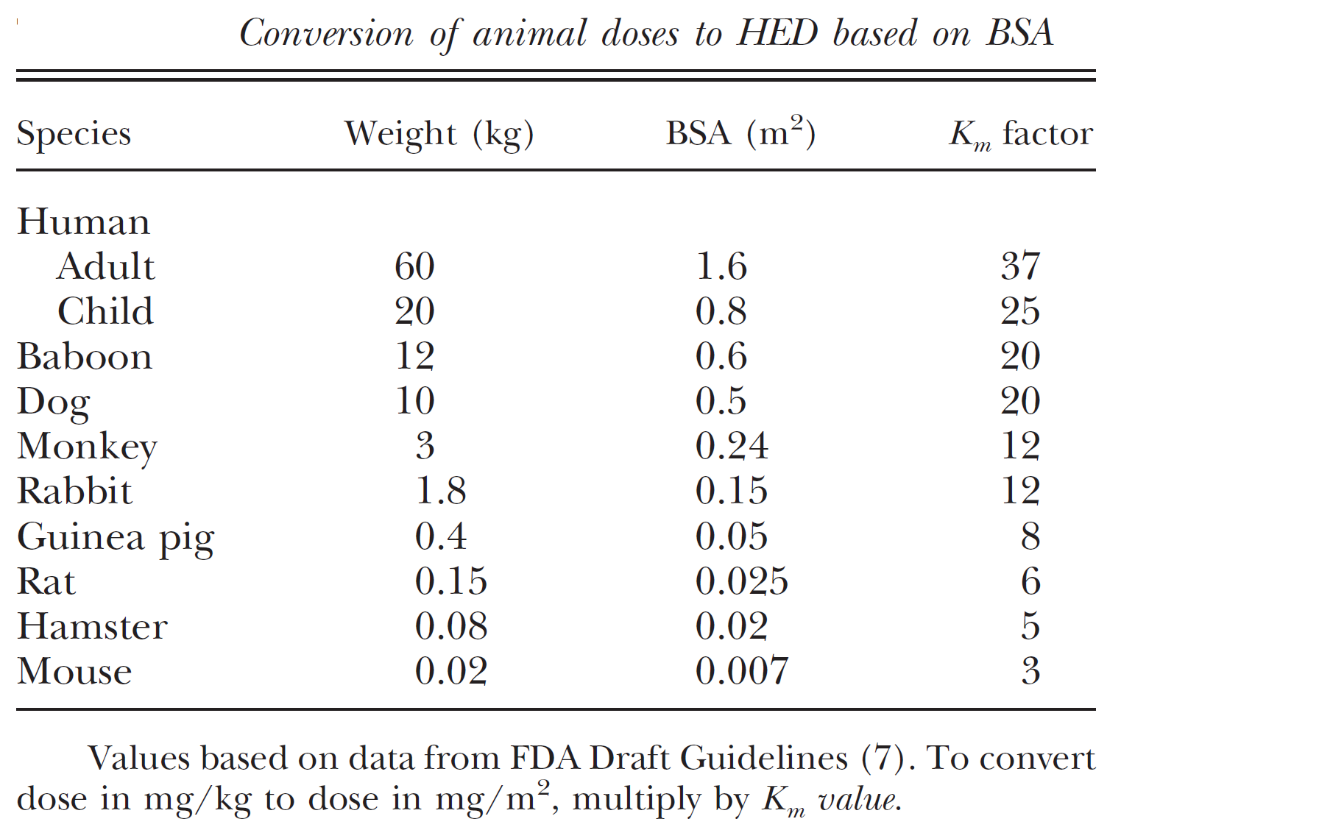


Based on the above dose translation formula, we arrived at the lowest human dose. This worked out to 5.6 mg of R and 560 ng of Cu (R:Cu ratio 1:10,000; wt for wt).

The next 2 higher doses were multiples of 10 of the lowest dose, i.e.,

50mg of R and 5µg of Cu (R:Cu ratio 1:10,000; wt for wt)

500mg of R and 50µg of Cu (R:Cu ratio 1:10,000; wt for wt).

Finally, the highest dose combination (4th dose) was based on that recommended in the over-the-counter resveratrol and copper as health supplements i.e., 500mg of R and 5mg of Cu (one capsule each of R and Cu).

In view of the fact that resveratrol dose of 50 mg with copper dose of 5mcg has not shown to better (see appendix 1 for clinical data) than resveratrol dose of 5.6 mg and copper 560 ng, the dose level of 5.6 mg reseveratrol and copper 560 ng will be expanded to accrue another 10 patients and further higher dose levels of R-Cu will not be explored.

- - 1. **Information on resveratrol and copper to be used in this study:**

Resveratrol and copper are available over-the-counter as health supplements. Trans-Resveratrol (Trade name – TransMax^TR^) is available as a health supplement in capsule form (500mg) and is approved for human use. It is available online from Biotivia LLC, USA and is recommended for oral use. The recommended human dose of Resveratrol as a health supplement is 500mg twice a day.

Copper (Trade name – Chelated Copper) for human use is available as health supplement in tablet form (5mg). It is available online from J.R. Carlson Laboratories Inc. USA and is recommended for oral use. The recommended human dose of Cu as a health supplement is 5mg once a day.

- - 1. **Preparation of resveratrol and copper:**

Transmax^TM^ (time release capsule of trans-resveratrol) and Chelated Copper^TM^ , available as 5mg tablets for human use, will be used for this study. Commercially available honey will be used as the vehicle for resveratrol, while copper will be dissolved in water at requisite concentration and administered concomitantly with resveratrol twice a day.

**Trans-Resveratrol:**

The exact amount of resveratrol will be weighed in butter paper and small packets will be prepared. The individual packets of 5.6 mg, 50 mg, 500 mg can be poured in to spoon and mixed with honey and swallowed.

**Chelated Copper:**

Copper will be administered as solution and will be prepared as follows:
**5 mg of Cu will be dissolved in 10 ml water**

**i.e. 0.5 mg / 1 ml of water**


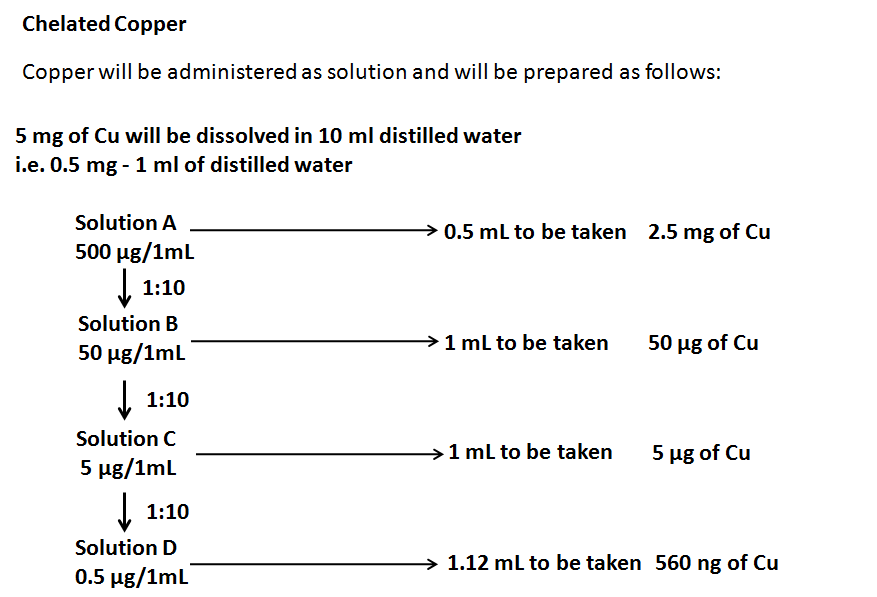


- - 1. **Administration of resveratrol (in honey) and copper (in water):**

Resveratrol and copper will be administered one after the other in the following 4-dose levels.

1. Only honey and water (1 tablespoon each twice a day) – first 5 patients
2. 5.6 mg of R and 560 ng of Cu (R:Cu ratio 1:10,000; wt for wt) – 6^th^ to 10^th^, patients.
3. 50mg of R and 5µg of Cu (R:Cu ratio 1:10,000; wt for wt) –11^th^ to 15^th^ patients.
4. 500mg of R and 50µg of Cu (R:Cu ratio 1:10,000; wt for wt) –16^th^ to 20^th^ patients.
5. 500mg of R and 5mg of Cu (one capsule each of R and Cu) –21^st^ to 25^th^ patients. This dose is the recommended for resveratrol and copper as health supplements.
   1. **Schedule of resveratrol-copper treatment**

The first dose of R-Cu would be administered 2 days (48 hours) prior to starting chemotherapy, immediately after the first blood sample collection and twice a day (approximately 12 hours apart), thereafter until day +21 (counting the day of stem cell infusion as day 0).

- 1. **Study Procedures and Monitoring of patients**

The first five of the 25 patients of BMT enrolled in this study will receive one tablespoon of honey and water, without copper or resveratrol, twice a day, starting 2 days prior to start of high dose conditioning chemotherapy until 21 days post-transplant, counting the day of stem cell infusion as day 0. The next 20 patients, 5 in each group, will receive 4 successively increasing dose-levels of resveratrol in honey and Cu in water, twice a day, starting 2 days prior to start of high dose conditioning chemotherapy until 21 days post–transplant, counting the day of stem cell infusion as day 0.

Keeping IEC informed of any untoward effects of R-Cu:

After accrual of each cohort of 5 patients at a dose-level of R-Cu, a committee comprised of all investigators will review the toxicity data to determine safety of proceeding to the next dose-level. These meetings will be minuted and the records will be submitted to IEC for information.

Standardizing the dose of chemotherapy in BMT patients:

All patients enrolling in this study would receive 200 mg/m^2^ of melphalan for patients of myeloma undergoing autologous transplant. Peripheral blood progenitor cells will be infused on day 0. Supportive care post transplant will be given as per standard guidelines. R-Cu will be given from 2 days prior to starting high dose conditioning regimen until day +21, counting the day of stem cell infusion as day 0.

All patients will be monitored for the following post-transplant complications:

- - 1. All grades of oral mucositis and duration of grade 3-4 oral mucositis
    2. All grades of diarrhea and duration of grade 3-4 diarrhea.
    3. All grades of nausea and vomiting and duration of grade 3-4 nausea and vomiting
    4. Duration of grade 4 neutropenia (i.e. ANC < 0.5 x 10^9^/L)
  1. **PK sampling and measurements –**

PK sampling to be performed on Day 7 of R-Cu dosing. Sampling times will vary based on the enrollment sequence of the subjects. Sparse sampling (5 samples per subject) will be performed for each subject.

The five subjects at each dose level will be divided into 3 groups for the purpose of PK sampling. For subjects 1 and 4 of each dose level, sampling will be performed as per GROUP 1 timings. Subjects 2 and 5 will be sampled as per GROUP 2 timings and subject 3 will be assigned to GROUP 3.

The sampling time points of the 3 groups are mentioned below:

Table 3: PK sampling protocol.

|  | GROUP 1 | GROUP 2 | GROUP 3 |
| --- | --- | --- | --- |
| Trough | √ | √ | √ |
| 0.5 | √ |  |  |
| 1 |  | √ |  |
| 2 |  |  | √ |
| 3 | √ |  |  |
| 4 |  | √ |  |
| 5 |  |  | √ |
| 7 | √ |  |  |
| 8 |  | √ |  |
| 10 |  |  | √ |
| 12 | √ | √ | √ |

A validated LC-MS/MS method will be used to determine plasma levels of Resveratrol with suitable modifications to the technique described by Muzzio et al.**^20^** Cu levels will be estimated using inductively coupled mass spectrometry (ICP-MS) at BARC, Mumbai.

- 1. **Informed Consent**

Patients included in the study will be counseled and would be asked to sign the IEC approved consent form.

1. **Blood and saliva collection**

Blood and saliva will be collected (8ml blood and 7 ml saliva), 48 hours prior to starting conditioning chemotherapy and then every 72 hours until 21 days post-transplant (days 0, 3, 6, 9, 12, 15, 18, 21), counting the day of stem cell infusion as day 0. R-Cu will be started 2-days prior to chemotherapy immediately after the first blood sample collection.

5ml blood will be collected in plain tubes, whereas 3ml will be collected in EDTA tubes (See Annexure 2). Serum will be separated from 5ml blood for cytokines, chromatin and lipid assay. 3ml blood collected in EDTA tubes will be used for TLC and PBMCs investigations. For pharmacokinetic studies, additional 3mL blood will be collected at the following time points: pre-dose, three samples at varying time-points between 0.5-10 hr and 12.0 hours on day 7 in 3mL EDTA tubes using sparse sampling strategy (refer PK sampling schedule – pg. 34). Blood collection chart is given in Annexure 2.

1. **Cytokine measurements**

The following cytokines will be estimated in blood and saliva: PGE2, TNF-α, INF-γ, IL-6, IL-1 and CRP. Plasma and saliva levels of cytokines will be measured by enzyme-linked immunoassay (ELISA).

1. **PBMCs investigations to estimate DNA damage and apoptosis**

The following indicators of DNA-damage (γ-H2AX), apoptosis (active Caspase-3) and inflammatory cytokines (NFκB and IL-6) will be estimated on PBMCs prepared from EDTA blood samples.

1. **Chromatin level estimation**

Chromatin in serum will be quantified by using the Cell Death Detection ELISA^plus^ kit (Roche Diagnostics GmbH, Germany). The assay is based on the quantitative sandwich-enzyme-immunoassay principle using mouse monoclonal antibodies directed against both DNA and histones. The absorbance kinetics will be measured at 405 nm by SPECTROstar Nano ELISA plate reader (BMG Labtech, Germany) and expressed as Arbitrary Units /mL.

1. **TLC estimation**

Total leukocyte count in blood will be estimated at each time-point when blood is withdrawn using standard procedures.

1. **Lipid profile estimation**

A complete lipid profile would be estimated by the Biochemistry Department at each time-point when blood is withdrawn using autoanalyzer. The profile would include: 1) total cholesterol; 2) triglycerides; 3) low-density lipoprotein (LDL) and 4) high-density lipoprotein (HDL).

1. **Statistical analysis plan**

Since this study is an exploratory pilot study aimed primarily at determining a signal of activity of R-Cu administration in reducing inflammatory cytokines and toxic side-effects of chemotherapy, formal plan for power analysis is not contemplated and any comparisons by tests of significance can only be considered preliminary.

The analytical plan is as follows:

CYTOKINE MEASUREMENT

1. Blood and saliva levels of each cytokine (6 in number) at each time point and for each dose-levels of R-Cu (5 in number), will be descriptively summarized using mean, standard deviation, standard error and 95% CI.
2. Cytokine levels (mean +/- SD) will be plotted for each cytokine and for each dose level of R-Cu over time.
3. The effect of various dose levels of R-Cu on each cytokine time-plot will be compared using repeated measures ANOVA.
4. Steps ‘1’, ‘2’ and ‘3’ above will be carried out for both serum and saliva separately.

PBMCs INVESTIGATIONS

The following indicators of DNA-damage (γ-H2AX), apoptosis (active Caspase-3) and inflammatory cytokines (NFκB and IL-6) will be analyzed on PBMCs prepared from EDTA blood samples.

SERUM CHROMATIN LEVELS

Analyses will be carried out for serum chromatin levels in the same way as for cytokine levels in steps 1-4, as stated above.

TLC AND LIPID PROFILE

Analyses will be carried out for TLC and lipid profile in the same way as for cytokine levels in steps 1-4, as stated above.

TRANSPLANT TOXICITY

1. For each Dose Level, various toxicities will be descriptively summarized (proportions) for grade 3-4 and all grades. The most important toxicities to be thus (grade 3-4) summarized are oral mucositis, diarrhea, nausea/vomiting, neutropenia and thrombocytopenia. No attempt will be made to compare the various dose levels with formal tests of significance for these proportions.
2. For each Dose Level, the durations of various grade 3-4 toxicities will be descriptively summarized as means, standard deviations and medians. No attempt will be made to compare the various dose levels with formal tests of significance for these means or medians by parametric or non-parametric tests.

PHARMACOKINETICS

The PK samples will be analyzed for the following parameters:

1. PK parameter calculations: We will use NON LINEAR MIXED EFFECTS MODELING for analysis of PK parameters using the NONMEM software (ICON Solutions, USA). Covariate effects, between subject variability and the residual variability will be explored. The PK parameters that will be calculated are Volume of Distribution (Vd), Clearance (Cl), Absorption and elimination rate constants (Ka, Kel). Using these primary PK parameters, secondary PK parameters such as Area Under the Curve (AUC), steady state concentration and half-life (t_1/2_) will be calculated.
2. Dose linearity assessment: Dose linearity will be assessed to check the linear increase in concentrations with increase of dose. This will be calculated by dose normalizing the PK parameters and overlapping the resultant normalized ratios at each dose range over each other.
3. Pharmacokinetic-Pharmacodynamic modelling: The Cytokine levels and the mucositis reductions measured from D1-7 will be incorporated into a Pharmacodynamic model. Primary PD parameters estimated will be Emax and EC50. Furthermore, this PD model will be linked with the PK model created earlier to estimate the dose response correlation. A soft link model with bidirectional flow of information between PK and PD models will be created.

Simulations: Based on the PKPD linked model created, simulations will be performed to identify the optimal dose. These simulations will be useful in identifying the dose and sample size for the next study.

1. **References:**
2. Burns J, Yokota T, Ashihara H, Lean ME, Crozier A. (2002). Plant foods and herbal sources of resveratrol. J Agric Food Chem. 50: 3337-3340.
3. Hector KL, Lagisz M, Nakagawa S. (2012). The effect of resveratrol on longevity across species: a meta-analysis. Biol Lett. 8: 790-793. doi: 10.1098/rsbl.2012.0316.
4. Suh DH, Kim Mi-K, Kim HS, Chung HH, Song YS. (2013). Cancer-specific Therapeutic Potential of Resveratrol: Metabolic Approach against Hallmarks of Cancer. Journal of FFHD. 3: 332-343. http://functionalfoodscenter.net/files/73514409.pdf
5. Pollack RM, Crandall JP. (2013). Resveratrol: therapeutic potential for improving cardiometabolic health. Am J Hypertens. 26: 1260-1268. doi: 10.1093/ajh/hpt165.
6. Menard C, Bastianetto S, Quirion R. (2013). Neuroprotective effects of resveratrol and epigallocatechin gallate polyphenols are mediated by the activation of protein kinase C gamma. Front Cell Neurosci. 7:281. doi: 10.3389/fncel.2013.00281.
7. Bhatt JK, Thomas S, Nanjan MJ. (2012). Resveratrol supplementation improves glycemic control in type 2 diabetes mellitus. Nutr Res. 32: 537-541. doi: 10.1016/j.nutres.2012.06.003.
8. Das S, Das DK. (2007). Anti-inflammatory responses of resveratrol. Inflamm Allergy Drug Targets. 6: 168-173.
9. Ahmad A, Farhan Asad S, Singh S, Hadi SM. (2000). DNA breakage by resveratrol and Cu(II): reaction mechanism and bacteriophage inactivation. Cancer Lett. 154: 29-37.
10. Leonard SS, Xia C, Jiang BH, Stinefelt B, Klandorf H, Harris GK, Shi X. (2003). Resveratrol scavenges reactive oxygen species and effects radical-induced cellular responses. Biochem Biophys Res Commun. 309: 1017-1026.
11. Gusman J, Malonne H, Atassi G. (2001). A reappraisal of the potential chemopreventive and chemotherapeutic properties of resveratrol. Carcinogenesis. 22: 1111-1117.
12. Lagouge M, Argmann C, Gerhart-Hines Z, Meziane H, Lerin C, Daussin F, Messadeq N, Milne J, Lambert P, Elliott P, Geny B, Laakso M, Puigserver P, Auwerx J. (2006). Resveratrol improves mitochondrial function and protects against metabolic disease by activating SIRT1 and PGC-1alpha. Cell. 127: 1109-1122.
13. Ponzo V, Soldati L, Bo S. (2014). Resveratrol: a supplementation for men or for mice? J Transl Med. 12:158. doi: 10.1186/1479-5876-12-158.
14. Walle T, Hsieh F, DeLegge MH, Oatis JE Jr, Walle UK. (2004). High absorption but very low bioavailability of oral resveratrol in humans. Drug Metab Dispos. 32: 1377-1382.
15. Li Y, Kuppusamy P, Zweier JL, Trush MA. (1995). ESR evidence for the generation of reactive oxygen species from the copper-mediated oxidation of the benzene metabolite, hydroquinone: role in DNA damage. Chem Biol Interact. 94: 101-120.
16. Fukuhara K, Miyata N. (1998). Resveratrol as a new type of DNA-cleaving agent. Bioorg Med Chem Lett. 8: 3187-3192.
17. Fukuhara K, Nagakawa M, Nakanishi I, Ohkubo K, Imai K, Urano S, Fukuzumi S, Ozawa T, Ikota N, Mochizuki M, Miyata N, Okuda H. (2006). Structural basis for DNA-cleaving activity of resveratrol in the presence of Cu(II). Bioorg Med Chem. 14: 1437-1443.
18. Azmi AS, Bhat SH, Hadi SM. (2005). Resveratrol-Cu(II) induced DNA breakage in human peripheral lymphocytes: implications for anticancer properties. FEBS Lett. 579: 3131-3135.
19. Hadi SM, Ullah MF, Azmi AS, Ahmad A, Shamim U, Zubair H, Khan HY. (2010). Resveratrol mobilizes endogenous copper in human peripheral lymphocytes leading to oxidative DNA breakage: a putative mechanism for chemoprevention of cancer. Pharm Res. 27: 979-988. doi: 10.1007/s11095-010-0055-4.
20. Subramaniam S, Vohra I, Iyer A, Nair NK, Mittra I. (2015). A paradoxical relationship between Resveratrol and copper (II) with respect to degradation of DNA and RNA. F1000Res. 4: 1145. doi: 10.12688/f1000research.7202.2.
21. Muzzio M, Huang Z, Hu SC, Johnson WD, McCormick DL, Kapetanovic IM. (2012). Determination of resveratrol and its sulfate and glucuronide metabolites in plasma by LC-MS/MS and their pharmacokinetics in dogs. J Pharm Biomed Anal. 59: 201-208. doi: 10.1016/j.jpba.2011.10.023.
22. Almeida L, Vaz‐da‐Silva M, Falcao A, Soares E, Costa R, Loureiro AI, Fernandes‐Lopes C, Rocha JF, Nunes T, Wright L, and Soares‐da‐Silva P. ‘Pharmacokinetic and safety profile of transresveratrol in a rising multiple‐dose study in healthy volunteers’*, Mol Nutr Food Res*. 2009; 53 Suppl 1: S7‐15
23. Boocock, D.J., Faust, G.E., Patel, K.R., Schinas, A.M., Brown, V.A., Ducharme, M.P., Booth, T.D., Crowell, J.A., Perloff, M., Gescher, A.J., Steward, W.P. and Brenner, D.E. (2007), ‘Phase I dose escalation pharmacokinetic study in healthy volunteers of resveratrol, a potential cancer chemopreventive agent’, *Cancer Epidemiol. Biomarkers Prev.*, vol.16, no.6, pp.1246–52
24. Brown VA, Patel KR, Viskaduraki M, Crowell JA, Perloff M, Booth TD, Vasilinin G, Sen A, Schinas AM, Piccirilli G, Brown K, Steward WP, Gescher AJ, and Brenner DE. ‘Repeat dose study of the cancer chemopreventive agent resveratrol in healthy volunteers: safety, pharmacokinetics, and effect on the insulin-like growth factor axis*’, Cancer Res*. 2010; 70: 9003‐9011.
25. Burkon A and Somoza V. ‘Quantification of free and proteinbound trans‐resveratrol metabolites and identification of transresveratrol‐ C/O‐conjugated diglucuronides ‐ two novel resveratrol metabolites in human plasma’*, Mol Nutr Food Res*. 2008; 52: 549‐557.
26. La Porte C, Voduc N, Zhang G, Seguin I, Tardiff D, Singhal N, and Cameron DW. ‘Steady‐State pharmacokinetics and tolerability of trans‐resveratrol 2000 mg twice daily with food, quercetin and alcohol (ethanol) in healthy human subjects’*, Clin Pharmacokinet*. 2010; 49: 449‐454.
27. Nunes T, Almeida L, Rocha JF, Falcao A, Fernandes‐Lopes C, Loureiro AI, Wright L, Vaz‐da‐Silva M, and Soares‐da‐Silva P. ‘Pharmacokinetics of trans‐resveratrol following repeated administration in healthy elderly and young subjects’*, J Clin Pharmacol*. 2009; 49: 1477‐1482.
28. Patel KR, Brown VA, Jones DJ, Britton RG, Hemingway D, Miller AS, West KP, Booth TD, Perloff M, Crowell JA, Brenner DE, Steward WP, Gescher AJ, and Brown K. ‘Clinical pharmacology of resveratrol and its metabolites in colorectal cancer patients’, *Cancer Res*. 2010; 70: 7392‐7399.
29. Ortuño J, Covas MI, Farre M, Pujadas M, Fito M, Khymenets O, Andres‐Lacueva C, Roset P, Joglar J, and RM L‐R. ‘Matrix effects on the bioavailability of resveratrol in humans’*, Food Chem*. 2010; 120: 1123‐1130.
30. Walle T, Hsieh F, DeLegge MH, Oatis JE, Jr., and Walle UK. ‘High absorption but very low bioavailability of oral resveratrol in humans’*, Drug Metab Dispos*. 2004; 32: 1377‐1382.
31. Goldberg DM, Yan J, and Soleas GJ. ‘Absorption of three wine‐related polyphenols in three different matrices by healthy subjects*’, Clin Biochem*. 2003; 36: 79‐87.
32. Ghanim H, Sia CL, Abuaysheh S, Korzeniewski K, Patnaik P, Marumganti A, Chaudhuri A, and Dandona P. ‘An antiinflammatory and reactive oxygen species suppressive effects of an extract of Polygonum cuspidatum containing resveratrol’*, J Clin Endocrinol Metab*, 2010; 95: E1‐8.
33. Ghanim H, Sia CL, Korzeniewski K, Lohano T, Abuaysheh S, Marumganti A, Chaudhuri A, and Dandona P. ‘A resveratrol andpolyphenol preparation suppresses oxidative and inflammatory stress response to a high‐fat, high‐carbohydrate meal’*,J Clin Endocrinol Metab*. 2011; 96: 1409‐1414.
34. Kennedy DO, Wightman EL, Reay JL, Lietz G, Okello EJ, Wilde A, and Haskell CF. ‘Effects of resveratrol on cerebral blood flow variables and cognitive performance in humans: a double‐blind, placebo‐controlled, crossover investigatio’,*. Am J Clin Nutr*,2010; 91: 1590‐1597.
35. Wong RH, Howe PR, Buckley JD, Coates AM, Kunz I, and Berry NM. ‘Acute resveratrol supplementation improves flow mediated dilatation in overweight/obese individuals with mildly elevated blood pressure*’, Nutr Metab Cardiovasc Dis*. 2010
36. Nguyen AV, Martinez M, Stamos MJ, Moyer MP, Planutis K, Hope C, and Holcombe RF. ‘Results of a phase I pilot clinical trial examining the effect of plant‐derived resveratrol and grape powder on Wnt pathway target gene expression in colonic mucosa and colon cancer*’,Cancer Manag Res*. 2009; 1: 25‐37.
37. Chow HH, Garland LL, Hsu CH, Vining DR, Chew WM, Miller JA, Perloff M, Crowell JA, and Alberts DS. ‘Resveratrol modulates drug‐ and carcinogen‐metabolizing enzymes in a healthy volunteer study’*, Cancer Prev Res (Phila).* 2010; 3: 1168‐1175
38. Elliott PJ, Walpole S, Morelli L, Lambert PD, Lunsmann W, Westphal CH, and Lavu S. ‘Resveratrol/SRT‐50’; *Drugs Fut*. 2009; 34: 291‐295. doi: 10.1358/dof.2009.034.04.1360696
39. Brasnyo P, Molnar GA, Mohas M, Marko L, Laczy B, Cseh J, Mikolas E, Szijarto IA, Merei A, Halmai R, Meszaros LG, Sumegi B, and Wittmann I. ‘Resveratrol improves insulin sensitivity, reduces oxidative stress and activates the Akt pathway in type 2 diabetic patients’*, Br J Nutr*. 2011: 1‐7.
40. Timmers S, Konings E, Bilet L, Houtkooper RH, van de Weijer T, Goossens GH, Hoeks J, van der Krieken S, Ryu D, Kersten S,Moonen‐Kornips E, Hesselink MK, Kunz I, Schrauwen‐Hinderling VB, Blaak EE, Auwerx J, et al. ‘Calorie restriction‐like effects of 30 days of resveratrol supplementation on energy metabolism and metabolic profile in obese humans’*, Cell Metab*. 2011; 14: 612‐ 622.
41. Crandall JP, Oram V, Trandafirescu G, Reid M, Kishore P, Hawkins M, Cohen HW, and Barzilai N. ‘Pilot Study of Resveratrol in Older Adults With Impaired Glucose Tolerance’; *J Gerontol A Biol Sci Med Sci*. 2012.
42. Popat R, Plesner T, Davies F, Cook G, Cook M, Elliott P, Jacobson E, Gumbleton T, Oakervee H, Cavenagh J. A phase 2 study of SRT501 (resveratrol) with bortezomib for patients with relapsed and or refractory multiple myeloma. Br J Haematol. 2013; 160: 714-717. doi: 10.1111/bjh.12154.
43. Fitzgerald DJ. Safety guidelines for copper in water. Am J Clin Nutr. 1998; 67(5 Suppl):1098S-1102S.
44. Food and Nutrition Board, Institute of Medicine. Copper. Dietary reference intakes for vitamin A, vitamin K, boron, chromium, copper, iodine, iron, manganese, molybdenum, nickel, silicon, vanadium, and zinc. Washington, D.C.: National Academy Press; 2001: pp224-257.
45. Turnlund JR, Jacob RA, Keen CL, Strain JJ, Kelley DS, Domek JM, Keyes WR, Ensunsa JL, Lykkesfeldt J, Coulter J. Long-term high copper intake: effects on indexes of copper status, antioxidant status, and immune function in young men. Am J Clin Nutr. 2004; 79: 1037-1044.
46. Turnlund JR, Keyes WR, Kim SK, Domek JM. Long-term high copper intake: effects on copper absorption, retention, and homeostasis in men. Am J Clin Nutr. 2005; 81: 822-828.
47. Kessler H, Bayer TA, Bach D, Schneider-Axmann T, Supprian T, Herrmann W, Haber M, Multhaup G, Falkai P, Pajonk FG. Intake of copper has no effect on cognition in patients with mild Alzheimer's disease: a pilot phase 2 clinical trial. J Neural Transm (Vienna). 2008; 115: 1181-1187. doi: 10.1007/s00702-008-0080-1.
48. Sonis S.Pathobiology of mucositis. Nat Can Rev.2004;4:277-284
49. Reagan-Shaw S, Nihal M, Ahmad N. Dose translation from animal to human studies revisited. FASEB J. 2008; 22: 659-661.

**Annexure 1**

**ACTREC CURCUMIN STUDY**

We conducted a pharmacokinetic – pharmacodynamic pilot study to explore the role of curcumin lozenges in decreasing inflammatory cytokines in serum and saliva in patients undergoing autologous transplant for multiple myeloma. The premise on which this study was conducted was based on the fact that inflammatory cytokines are involved in pathogenesis of mucositis through the NF –kappa pathway and curcumin inhibits the NF- kappa pathway.Therefore, in this study ,initial 10 patients did not receive curcumin but the levels of serum and salivary inflammatory cytokines were measured at predetermined time points prior to administration of high dose melphalan and then on every Monday , Wednesday and Friday till day +14 post transplant and then on day +28.The cytokines measured were The serum and salivary cytokines measured were IL-1,IL-6, IL-8, IL-17,TNF alpha, TGF beta, PGE2 and interferon gamma.

The subsequent 27 patients received curcumin lozenges , 400 mg twice daily, 2 days prior to administration of melphalan till day 28 post transplant. Trough curcumin levels were also measured weekly post transplant. The median levels of various cytokines in serum and saliva over 28 days post transplant were compared in the 2 groups as shown below:

- The median serum levels of interferon gamma **(IFN-γ)** were reduced in the curcumin group on day+13 and day+28 (P=0.067 and 0.040 respectively) compared to the control group (figure-1). The median salivary INF- γ levels were similar between the two groups (figure-2).

Figure-1:- Median serum INF-γ levels at each time point between the two groups.

Figure-2:- Median salivary INF-γ levels at each time point between the two groups.

- The interleukin-6 (**IL-6**) levels in serum were significantly higher in the curcumin group on day-3, day+1 and day+6 as shown in figure-3 (P= 0.078, 0.005 and 0.001 respectively). Also the salivary levels were significantly higher on day-3 and day+1 (figure-4; P=0.040 and 0.044 respectively). .

**IL6- Serum**

P value

Day-1=0.078

Day+1=0.005

Day+6=0.001

Figure-3:- Median serum IL-6 levels at each time point between the two groups.

P value

Day-1=0.040

Day+1=0.044

Figure-4:- Median salivary IL-6 levels at each time point between the two groups.

- Median serum Tumor Necrosis factor-alpha (**TNF-α**) levels were higher in the curcumin group on day+8 (figure-5; P=0.084). The median salivary levels were also higher on day+1, day+13 and day+28 in the curcumin group (figure-6; P=0.019, 0.017 and 0.003 respectively).

P value

Day+8:- 0.084

Figure-5:- Median serum TNF-α levels at each time point between the two groups.

P value

Day+1=0.019

Day+13=0.017

Day+28=0.003

Figure-6:- Median salivary TNF-α levels at each time point between the two groups.

- Serum Transforming growth factor – beta (**TGF-β**) levels were high in the curcumin group on day-1 and day0 (figure-7; P=0.044 and 0.048 respectively). Median salivary levels were similar in both groups (figure-8).

P value

Day-1=0.044

Day 0=0.048

Figure-7:- Median serum TGF-β levels at each time point between the two groups.

P value

Day+1=0.048

Figure-8:- Median salivary TGF-β levels at each time point between the two groups.

- The serum levels of interleukin-1 (**IL-1**) were not detectable in both the non-curcumin and non-curcumin groups. Median salivary level on day+1 was significantly higher in the curcumin group (figure-9; P=0.048).

Figure-9:- Median salivary IL-1 at each time point between the two groups.

- Interleukin-8 (**IL-8**) levels in serum of the patients in the curcumin group, initially was high on day+6, but gradually decreased and became significantly low by day+28 compared to the non-curcumin group (figure-10; P=0.013 and 0.02 respectively). The median salivary levels on day+4, day+6, day+8, day+11 and day+13 were significantly low in the curcumin group when compared to the control group (figure-11; P=0.004, 0.009, 0.024, 0.056 and 0.009 respectively).

P value

Day+6=0.013

Day+28=0.020

Figure-10:- Median serum IL-8 levels at each time point between the two groups.

P value

Day+4=0.004

Day+6=0.009

Day+8=0.024

Day+11=0.05

Day+13=0.009

Figure-11:- Median salivary IL-8 levels at each time point between the two groups.

- Median serum interleukin-17 (**IL-17**) levels were not detectable in both groups of patients. In the salivary samples, the levels of IL-17 were only detected in the control group (figure-12), but did not show a statistically significant difference between the two groups.

Figure-12:- Median salivary IL-17 levels at each time point between the two groups.

- Prostaglandin E2 (**PGE2**) levels were measured in the initial 20 patients of the pilot study due to logistic issues so far. In the curcumin group, the median serum levels of PGE2 were significantly reduced on day+6, day+8, day+11 and day+13 (figure-13; P=0.035, 0.053, 0.017and 0.013 respectively) compared to control group. The salivary levels were similar in both groups (figure-14).

P value

Day+6=0.035

Day+8=0.053

Day+11=0.017

Day+13=0.013

Figure-13:- Median serum PGE2 levels at each time point between the two groups.

Figure-14:- Median salivary PGE2 levels at each time point between the two groups.

In the above study, we also compared the incidence and maximum grade of various toxicities in both control and curcumin groups as shown in the table 1 below.

| Table-1:- Toxicity profile | | | |
| --- | --- | --- | --- |
| Toxicity | Run-in Phase  (Non curcumin group)  n=10 | Treatment Phase  (Curcumin group)  n=27 | P value |
| Incidence of grade 3-4 oral mucositis  (%) | 6  (60%) | 12  (44%) | 0.4756 |
| Incidence of any grade of mucositis  (%) | 10  (100%) | 26  (96%) | 1.0 |
| Median duration of grade 3-4 oral mucositis  (days) | 5.5  (2 – 9) | 3.5  (1 – 7) | 0.125 |
| Incidence of grade 3-4 diarrhea  (%) | 7  (70%) | 9  (33%) | 0.067 |
| Incidence of any grade of diarrhea  (%) | 10  (100%) | 27  (100%) | 1.0 |
| Median duration of grade 3-4 diarrhea  (days) | 2  (1 – 7) | 5  (2 – 9) | 0.091 |
| Incidence of grade 3-4 vomiting  (%) | 4  (40%) | 1  (3.7%) | 0.0136 |
| Incidence of any grade of vomiting  (%) | 6  (60%) | 14  (52%) | 0.7246 |
| Incidence of use of opiod analgesics  (%) | 5  (50%) | 10  (37%) | 0.7076 |
| Incidence of use of TPN  (%) | 9  (90%) | 14  (51%) | 0.0456 |
| Median duration of use of TPN  (days) | 9  (4 – 16) | 7  (4 – 12) | 0.159 |
| Median duration of hospital stay  (days) | 18  (16 – 22) | 19  (15 – 26) | 0.623 |

**Annexure 2**

Blood collection chart for R-Cu BMT study:

| Time period | Cytokines | CDDE | Lipid profile | TLC / CBC / PBMCs | PK studies |
| --- | --- | --- | --- | --- | --- |
|  | 3 mL | 1 mL | 1 mL | 3 mL | 3 mL |
|  | Plain tube | Plain tube | Plain tube | EDTA tube | EDTA tube |
| - 48 hr | **✓** | **✓** | **✓** | **✓** | **X** |
| + 24 hr | **✓** | **✓** | **✓** | **✓** | **X** |
| + 96 hr | **✓** | **✓** | **✓** | **✓** | **X** |
| +168 hr | **✓** | **✓** | **✓** | **✓** | **✓** |
| + 168.5 hr  to  +178 hr | **X** | **X** | **X** | **X** | **✓**  **(3 samples)** |
| + 180 hr | **X** | **X** | **X** | **X** | **✓** |
| + 240 hr | **✓** | **✓** | **✓** | **✓** | **X** |
| + 312 hr | **✓** | **✓** | **✓** | **✓** | **X** |
| + 384 hr | **✓** | **✓** | **✓** | **✓** | **X** |
| + 456 hr | **✓** | **✓** | **✓** | **✓** | **X** |
| + 528 hr | **✓** | **✓** | **✓** | **✓** | **X** |
| + 600 hr | **✓** | **✓** | **✓** | **✓** | **X** |
| + 672 hr | **✓** | **✓** | **✓** | **✓** | **X** |

**Annexure 3**

| **List of cytochrome substrates** | | |
| --- | --- | --- |
| **CYP3A4 Substrates** | **CYP2D6Substrates** | **CYP2C9 Substrates** |
| Immunosuppressants  [ciclosporin](https://en.wikipedia.org/wiki/Ciclosporin)  [tacrolimus](https://en.wikipedia.org/wiki/Tacrolimus)  [sirolimus](https://en.wikipedia.org/wiki/Sirolimus)  Chemotherapeutics  [docetaxel](https://en.wikipedia.org/wiki/Docetaxel)  [tamoxifen](https://en.wikipedia.org/wiki/Tamoxifen)  [paclitaxel](https://en.wikipedia.org/wiki/Paclitaxel)  [cyclophosphamide](https://en.wikipedia.org/wiki/Cyclophosphamide)[d](https://en.wikipedia.org/wiki/Doxorubicin)  [erlotinib](https://en.wikipedia.org/wiki/Erlotinib)  [etoposide](https://en.wikipedia.org/wiki/Etoposide)  [ifosfamide](https://en.wikipedia.org/wiki/Ifosfamide)  [teniposide](https://en.wikipedia.org/wiki/Teniposide)  [vinblastine](https://en.wikipedia.org/wiki/Vinblastine)  [vincristine](https://en.wikipedia.org/wiki/Vincristine)  [vindesine](https://en.wikipedia.org/wiki/Vindesine)  [imatinib](https://en.wikipedia.org/wiki/Imatinib)  [irinotecan](https://en.wikipedia.org/wiki/Irinotecan)  [sorafenib](https://en.wikipedia.org/wiki/Sorafenib)  [sunitinib](https://en.wikipedia.org/wiki/Sunitinib)  [vemurafenib](https://en.wikipedia.org/wiki/Vemurafenib)  [temsirolimus](https://en.wikipedia.org/wiki/Temsirolimus)  [anastrozole](https://en.wikipedia.org/wiki/Anastrozole)  [gefitinib](https://en.wikipedia.org/wiki/Gefitinib)  [azole antifungals](https://en.wikipedia.org/wiki/Azole_antifungal)  [ketoconazole](https://en.wikipedia.org/wiki/Ketoconazole)  [itraconazole](https://en.wikipedia.org/wiki/Itraconazole)  [macrolides](https://en.wikipedia.org/wiki/Macrolide_antibiotics)  [clarithromycin](https://en.wikipedia.org/wiki/Clarithromycin)  [erythromycin](https://en.wikipedia.org/wiki/Erythromycin)  [dapsone](https://en.wikipedia.org/wiki/Dapsone)  [opioids](https://en.wikipedia.org/wiki/Opioids) (mainly analgesics)  [alfentanil](https://en.wikipedia.org/wiki/Alfentanil)  [buprenorphine](https://en.wikipedia.org/wiki/Buprenorphine)  [codeine](https://en.wikipedia.org/wiki/Codeine)  [fentanyl](https://en.wikipedia.org/wiki/Fentanyl)  [methadone](https://en.wikipedia.org/wiki/Methadone)  [levacetylmethadol](https://en.wikipedia.org/wiki/Levacetylmethadol)  [tramadol](https://en.wikipedia.org/wiki/Tramadol)  [benzodiazepines](https://en.wikipedia.org/wiki/Benzodiazepines)  [alprazolam](https://en.wikipedia.org/wiki/Alprazolam)  [midazolam](https://en.wikipedia.org/wiki/Midazolam)  [diazepam](https://en.wikipedia.org/wiki/Diazepam)  [statins](https://en.wikipedia.org/wiki/Statin)  [atorvastatin](https://en.wikipedia.org/wiki/Atorvastatin)  [lovastatin](https://en.wikipedia.org/wiki/Lovastatin)  [simvastatin](https://en.wikipedia.org/wiki/Simvastatin)  [calcium channel blockers](https://en.wikipedia.org/wiki/Calcium_channel_blockers)  [nifedipine](https://en.wikipedia.org/wiki/Nifedipine)  [verapamil](https://en.wikipedia.org/wiki/Verapamil)  [amlodipine](https://en.wikipedia.org/wiki/Amlodipine)  [amiodarone](https://en.wikipedia.org/wiki/Amiodarone)  [quinidine](https://en.wikipedia.org/wiki/Quinidine)  [H1-receptor antagonists](https://en.wikipedia.org/wiki/H1-receptor_antagonist)  [terfenadine](https://en.wikipedia.org/wiki/Terfenadine)  [astemizole](https://en.wikipedia.org/wiki/Astemizole)  [chlorphenamine](https://en.wikipedia.org/wiki/Chlorphenamine)  [Protease inhibitors](https://en.wikipedia.org/wiki/Protease_inhibitors)  [indinavir](https://en.wikipedia.org/wiki/Indinavir)  [ritonavir](https://en.wikipedia.org/wiki/Ritonavir)  [saquinavir](https://en.wikipedia.org/wiki/Saquinavir)  [nelfinavir](https://en.wikipedia.org/wiki/Nelfinavir)  non-nucleoside [reverse transcriptase inhibitors](https://en.wikipedia.org/wiki/Reverse_transcriptase_inhibitors)  [nevirapine](https://en.wikipedia.org/wiki/Nevirapine)  some [glucocorticoids](https://en.wikipedia.org/wiki/Glucocorticoids)  [budesonide](https://en.wikipedia.org/wiki/Budesonide)  [hydrocortisone](https://en.wikipedia.org/wiki/Hydrocortisone)  [dexamethasone](https://en.wikipedia.org/wiki/Dexamethasone)  [aprepitant](https://en.wikipedia.org/wiki/Aprepitant)  [warfarin](https://en.wikipedia.org/wiki/Warfarin)  [omeprazole](https://en.wikipedia.org/wiki/Omeprazole) | All tricyclic antidepre- ssants  Most SSRIs  Opioids  codeine  tramadol  tapentadol  antipsychotics  haloperidol  risperidone  tamoxifen  beta-blockers  metoprolol  carvedilol  Class antiarrhythmics  Ondansetron  Tropisetron  chlorphenamine  dexfenfluramine  dextromethorphan  metoclopramide  • promethazine | NSAIDs  Celecoxib  Lornoxicam  Diclofenac  ibuprofen  naproxen  ketoprofen  piroxicam  meloxicam  phenytoin  fluvastatin  sulfonylureas  angiotensin II receptor antagonists  losartan  S-warfarin  terbinafine  amitriptyline  fluoxetine  nateglinide  rosiglitazone  tamoxifen  torasemide  ketamine  montelukast |

**Below is the summary of protocol amendments made during the course of the study**

**Summary List of Changes (from version 1 to version 2)**

| **Name of document** | **Revised version/Date** | **Section** | **Risk/Benefit Assessment /Justification** |
| --- | --- | --- | --- |
| Project protocol | Version 2 dated 31 August 2016 | 1) title, 2) hypothesis, 3) aims, 5) secondary objectives, 6) exploratory objectives, 7) background, 8) study population, 9) study intervention, 10) schedule of resveratrol-copper treatment, 11) study procedures and monitoring of patients, 12) PK sampling and measurements, 13) blood and saliva collection, 14) statistical analysis plan | Resveratrol has been extensively studied in clinical trials in humans in a range of therapeutic doses in healthy human volunteers as well as in patients with diabetes, obesity and cancer. The studies are summarized in Table no.1 below. Overall the data indicates that there are no major safety concerns with doses ranging up to 5000 mg/day. In some studies, higher doses caused mild gastrointestinal intolerance in a small fraction of patients.  In one study in myeloma patients, there were concerns about renal toxicity (Popat et al., Table 1); however, all patients of myeloma were at risk of this complication, the dose of resveratrol (5000 mg / day) was 5 times higher than our highest proposed dose and the formulation of resveratrol was a more orally bioavailable micronized version.  Given the above observations, it is reasonable to state that the dose levels prescribed in our study are highly unlikely to result in major toxicities. |

**Summary List of Changes (from version 2 to version 3)**

| **Name of document** | **Revised version/Date** | **Section** | **Change(s)** | **Risk/Benefit Assessment /Justification** |
| --- | --- | --- | --- | --- |
| Protocol | Version 3 dated November 2017 | Methodology:  Blood and saliva collection | Change in the timing of blood sample | There is no additional risk to the patient since the sample volume is the same |

**Summary List of Changes (from version 3 to version 4)**

| **Name of document** | **Revised version/Date** | **Section** | **Change(s)** | **Risk/Benefit Assessment /Justification** |
| --- | --- | --- | --- | --- |
| Protocol | Version 4 dated August 2018 | Methodology | Expand cohort 2 of this study to enroll another 10 patients at the dose level of 5.6 mg resveratrol and 560 ng of copper. | We have seen that dose level 2 of R-Cu (50 mg resveratrol and 5 mcg copper) is no better or may be slightly worse than the lower dose level of 5.6 mg resveratrol and 560 ng of copper. The clinical supporting data to this effect is shown in appendix 1.  Therefore, we feel that further higher dose levels will not be beneficial and the dose level of 5.6 mg resveratrol and 560 ng of copper would need to be expanded to accrue another 10 patients. This may give us a more robust data on R-Cu’s efficacy at the above dose level. |
